# Visualizing molecular interactions that determine assembly of a bullet-shaped vesicular stomatitis virus particle

**DOI:** 10.1038/s41467-022-32223-1

**Published:** 2022-08-15

**Authors:** Simon Jenni, Joshua A. Horwitz, Louis-Marie Bloyet, Sean P. J. Whelan, Stephen C. Harrison

**Affiliations:** 1 Department of Biological Chemistry and Molecular Pharmacology, Harvard Medical School, Boston, MA 02115 USA; 2 Department of Microbiology, Harvard Medical School, Boston, MA 02115 USA; 3 Department of Molecular Microbiology, Washington University in St. Louis, St. Louis, MO 63110 USA; 4 Howard Hughes Medical Institute, Harvard Medical School, Boston, MA 02115 USA; 5 Laboratory of Molecular Medicine, Boston Children’s Hospital, Boston, MA 02115 USA; 6 Present Address: Molecular Pharmacology and Virology Group, PureTech Health LLC, Boston, MA 02210 USA

**Keywords:** Cryoelectron microscopy, Transcriptional regulatory elements

## Abstract

Vesicular stomatitis virus (VSV) is a negative-strand RNA virus with a non-segmented genome, closely related to rabies virus. Both have characteristic bullet-like shapes. We report the structure of intact, infectious VSV particles determined by cryogenic electron microscopy. By compensating for polymorphism among viral particles with computational classification, we obtained a reconstruction of the shaft (“trunk”) at 3.5 Å resolution, with lower resolution for the rounded tip. The ribonucleoprotein (RNP), genomic RNA complexed with nucleoprotein (N), curls into a dome-like structure with about eight gradually expanding turns before transitioning into the regular helical trunk. Two layers of matrix (M) protein link the RNP with the membrane. Radial inter-layer subunit contacts are fixed within single RNA-N-M1-M2 modules, but flexible lateral and axial interactions allow assembly of polymorphic virions. Together with published structures of recombinant N in various states, our results suggest a mechanism for membrane-coupled self-assembly of VSV and its relatives.

## Abstract (web summary)

Vesicular stomatitis virus (VSV) is the most widely studied prototype for negative-sense RNA viruses. Structure determination of VSV particles is particularly challenging because they are polymorphic with different helical symmetries. Here, Jenni et al. apply computational classification approaches to sort different morphologies, and obtain CryoEM reconstructions at 3.5–4.1 A resolution. They show that the matrix protein (M) is present in the virion in two layers, of which the inner layer associates with N protein of vRNPs.

## Introduction

Non-segmented, negative-strand (NNS) RNA viruses of the order *Mononegavirales* include such human pathogens as Ebola virus, respiratory syncytial virus (RSV), measles virus, and rabies virus (RABV). The study of vesicular stomatitis virus (VSV), a prototypic mammalian rhabdovirus, has contributed to our fundamental understanding of the biology of NNS RNA viruses^1,2^. The viral genome comprises a regulatory 3′ terminal leader region and 5 genes in the following order: N (nucleoprotein), P (phosphoprotein), M (matrix protein), G (glycoprotein) and L (large polymerase), and a 5′ terminal trailer region. All of these proteins are present in mature, infectious virions, in which the negative-strand, genomic RNA is coated with the N protein (one N per 9 ribonucleotides) to form a ribonucleoprotein (RNP) complex^3^. The VSV RNP complex assembles, together with M, into a helical, bullet-shaped structure, as it buds through the plasma membrane of an infected cell^4^. Projecting from the membrane are trimeric glycoproteins (G), which mediate host cell attachment and low pH-induced membrane fusion^5–7^. Multiple copies of L, a polyfunctional RNA polymerase, are tethered by their P cofactor to the interior of the helical RNP. Using the RNP complex as template, the L protein catalyzes transcription and replication with its RNA-dependent RNA polymerase (RdRp) activity and also caps and methylates the 5′ ends of the viral mRNA transcripts^8,9^.

Atomic structures from x-ray crystallography and cryogenic electron microscopy (cryo-EM) are available for all five VSV proteins^3,5,6,10–16^. VSV N, expressed as a recombinant protein in bacterial cells, binds random fragments of RNA and forms ring-like assemblies^17,18^. A crystal structure of the predominant species, a decamer with up to 90 nucleotides of RNA, has been particularly informative for understanding the organization of the virion^3,19,20^. N is an elongated, two-lobed protein of ~420 amino-acid residues; the single stranded RNA binds in a cleft between the two lobes, with nine nucleotides per N subunit. Flexible extensions from each N subunit contact the neighboring subunits along the RNA. The N-terminal arm of each subunit (residues 4–19) embraces the C-terminal lobe of the subunit 5′ to it along the genomic RNA, folding into a shallow groove; an extended loop of the C-terminal lobe (residues 342–357) contacts the C-terminal lobe of the subunit 3′ to it.

Early examination of the RNP complex in intact bullet-shaped virions by EM of negatively stained VSV particles suggested a helix of about 30 coils with an external diameter of 490 Å^21^. The hemispherical tip of the bullet shape was proposed to arise from four additional turns with diminishing diameter. A more refined model was obtained from a cryo-EM reconstruction of the helical region of the virion, referred to in that work as the “trunk”, determined at about 10 Å resolution^22^. Docking of the N and M protein crystal structures into the density showed that the RNP complex forms an inner, left-handed helix, surrounded by a second, outer helix of M proteins. The long axis of the N protein is approximately perpendicular to the helix axis, with its RNA-binding cleft oriented towards the tip of the virion. The 3′ end of the genomic RNA is therefore at the domed end of the particle and the 5′ end, at the blunt end of the particle.

To reach higher resolution, we recorded cryo-EM images of intact VSV virions preserved in vitreous ice and used a supervised classification approach to sort virions with different morphologies. We obtained a helical reconstruction of the trunk at 4.1 Å resolution, and a local reconstruction of the basic building module (one N and two M proteins) at 3.5 Å resolution. Our atomic model of the helical nucleocapsid shows that two concentric layers of M, named M1 and M2, coat the helically folded RNP ribbon. The assembly is held together by invariant interactions between the N, M1, and M2 subunits within the basic module, and by contacts with neighboring modules through flexible extensions. We also obtained an asymmetric reconstruction of the domed tip at about 9 Å resolution. The RNP ribbon interacts with the viral membrane at the tip of the virion, curls into about eight turns with increasing diameter, at which point the ribbon transitions into the regular helical trunk.

## Results

### Cryo-EM structure determination of the VSV helical nucleocapsid

**Fig. 1:**
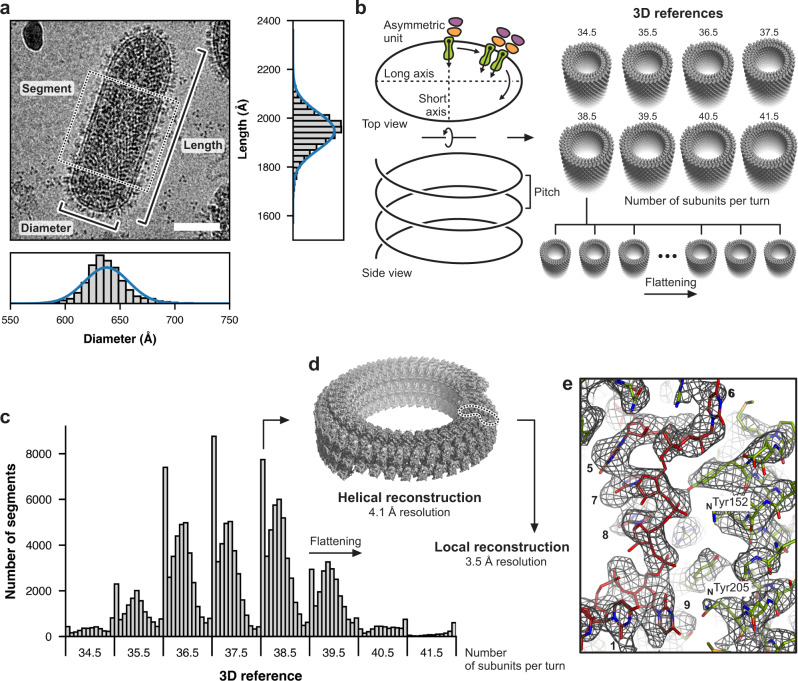
Cryo-EM analysis of the helical VSV nucleocapsid. **a** Cryo-EM image of a bullet-shaped vesicular stomatitis virus (VSV) particle. The image was low-pass filtered and contrast enhanced. The scale bar corresponds to 50 nm. The horizontal and vertical histograms show the distribution of measured diameter and length, respectively, from 5763 individual particles. Measurements were taken between the middle of the lipid bilayers as indicated and possible out-of-plane tilting was ignored. The solid blue lines are normal distribution fits to the histograms with means and standard deviations of 638 ± 20 Å for the diameter and 1947 ± 76 Å for the length. The dashed box shows one segment image extracted along the VSV trunk. **b** Preparation of 3D references for supervised classification. Density of one asymmetric unit initially obtained from a low resolution reconstruction comprising one N protein (green) and two M proteins (orange and purple) was expanded to create 3D references with different numbers of subunits per turn (34.5, 35.5, 36.5, 37.5, 38.5, 39.5, 40.5, and 41.5) and different degrees of flattening. **c** Histogram of the supervised classification. Each segment image was assigned to one of the 96 3D references based on the highest alignment score. **d** Helical reconstruction obtained from sorted segments that scored best with un-flattened or only moderately flattened 3D references. Shown here is the reconstruction calculated from segments with 38.5 subunits per turn. **e** Close-up view of the cryo-EM density map after local reconstruction of the asymmetric unit with a nominal resolution of 3.5 Å. Subparticles were aligned by alignment-by-classification and subsequent local alignment. RNA bases are numbered.

As in the published 10 Å resolution structure^22^, we did not detect regular features for the G protein, which is present in many fewer copies than N and which has no fixed position with respect to the internal assembly. We, therefore, concentrated entirely on the internal structures. For an initial reconstruction from trunk segments extracted from our cryo-EM images, we applied the previously reported helical symmetry (37.5 subunits per turn, 51.7 Å pitch)^22^. Despite the fact that the data had been acquired with a direct electron detector, the resolution of the reconstruction was only about 8 Å, suggesting structural heterogeneity among the extracted helical segments. When we measured the length (1947 ± 76 Å) and diameter (638 ± 20 Å) of the bullet-shaped particle projections in our micrographs, we found broad distributions (Fig. 1a) around the previously determined values (1960 ± 80 and 660 Å, respectively)^22^; another study had reported a similarly broad diameter distribution for reconstituted trunk segments^23^. Assembly of virions with longer or truncated RNA genomes could lead to particles with different length^21^. Moreover, variations in the number of nucleoprotein (N) and matrix protein (M) subunits per helical coil in the trunk, and/or flattening of the particles in vitreous ice during cryo-EM specimen preparation could explain the observed distributions in diameter. After conventional 2D classification of helical trunk segments, even the best-looking 2D class averages contained heterogeneous segments (Supplementary Fig. 1a). Top views of partially assembled or disrupted particles showed helical turns with different numbers of subunits (Supplementary Fig. 1b), and a scatter plot showed that bullet-shaped particles with a larger diameter were on average shorter (Supplementary Fig. 2a). These observations suggested that we would need to account for variations in the number of subunits per helical turn in order to improve the resolution of a cryo-EM reconstruction of the helical VSV nucleocapsid.

We therefore sorted helical trunk segments by supervised classification (see “Methods”). To do so, we prepared 3D references with different numbers of subunits per turn and different degrees of flattening, by expanding the asymmetric unit taken from the initial low-resolution map (Fig. 1b). We first globally aligned each helical segment to eight non-flattened 3D references with the number of subunits per turn, *N*, ranging from 34.5 to 41.5. Class assignment based on the highest alignment score correlated with the average measured particle length of the virions from which the segments had been extracted (Supplementary Fig. 2b). In a second step, we locally aligned the segments of each non-flattened class to flattened 3D references. Figure 1c shows the partitioning of segments into the final 96 classes. Helical reconstructions calculated from segments that belonged to unflattened classes showed substantially improved resolution, the best being the reconstruction obtained from the 38.5 subunits-per-turn class, which had an overall resolution of 4.1 Å as judged by Fourier shell correlation (FSC) analysis (Supplementary Table 1 and Supplementary Fig. 3a). Note that 38.5 subunits per coil were predicted based on the diameter of the decameric N-RNA ring in the crystal structure^3^ and the dimension of virions observed in EM images^21^.

We also calculated a local reconstruction by alignment and averaging of the asymmetric units (N subunit, 9 bound RNA nucleotides, and the two matrix protein subunits M1 and M2) from all *N* classes, yielding a nominal resolution of 3.5 Å (Fig. 1e and Supplementary Fig. 3b). This map was then used to build and refine a molecular model of the asymmetric unit (Supplementary Table 1). We modeled the RNA nucleotides as poly-uridine, because the density in our reconstruction is the average of different sequences. The model from the local reconstruction map was then also placed into the *N* = 38.5 helical reconstruction and refined at 4.1 Å resolution (Supplementary Table 1 and Supplementary Fig. 3c) and rigid-body fitted into the maps for the other *N* classes.

### Structure of the VSV helical nucleocapsid (RNA-N-M1-M2 helix)

**Fig. 2:**
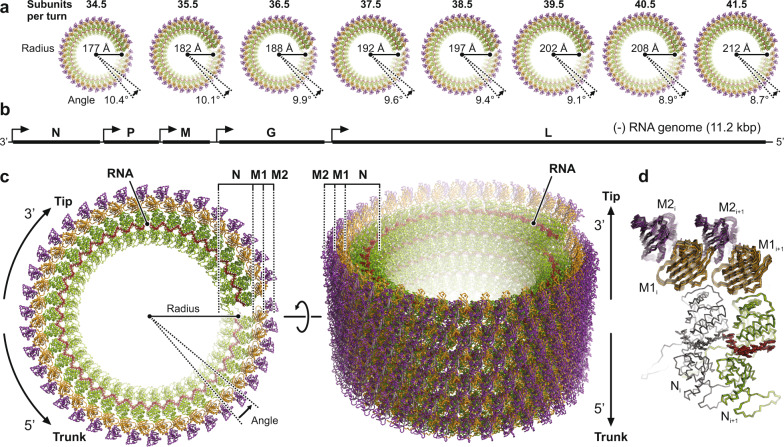
Structure of the helical VSV nucleocapsid. **a** Top views of the helical nucleocapsids from virions with different numbers of subunits per turn. The nucleoprotein (N) is colored green, the RNA red, and the two matrix proteins (M1 and M2) are colored orange and purple, respectively. **b** Schematic organization of the VSV negative-strand genomic RNA. Open reading frames encoding the five viral proteins are indicated by solid black lines. Transcription start sites are indicated by arrows. The 3′ end of the single-stranded RNA terminates at the tip of the virion, the 5′ at the base of the trunk. **c** Structure of the helical nucleocapsid with *N* = 38.5 in top (left) and side (right) view. Molecules are colored as in **a**. The three protein layers are labeled N (nucleoprotein), M1 (inner layer matrix protein), and M2 (outer layer matrix protein). **d** Structural comparison of the subunit arrangement in nucleocapsids with different *N*. Two adjacent asymmetric units (RNA-N-M1-M2) of each class are shown in ribbon representation and are superimposed on one N protein (colored in gray).

The asymmetric unit of the VSV helical nucleocapsid, which repeats every 9 RNA nucleotides, contains one nucleoprotein (N) subunit and two (chemically identical) matrix protein subunits (M1 and M2). Depending on the number of subunits per helical turn, the radius of the RNA strand from the center of the virion ranges from 177 Å (*N* = 34.5) to 212 Å (*N* = 41.5) (Fig. 2a), in close agreement with the position of the densely stained band at a radius of 175–205 Å seen in the early EM studies of negatively stained VSV particles^21^. From the length of the RNA genome (Fig. 2b) and the 9-nucleotide spacing, one would expect 1240 N proteins in a fully assembled virion, close to the value of 1258 determined by scanning transmission electron microscopy (STEM) and biochemical analysis^24^. If one subtracts approximately 220 N subunits that form the bullet tips up to the point where the RNP ribbon transitions into a regular helix (see below), the number of coils in the helical part of the VSV nucleocapsid is 24.6–29.6, with 26.5 in case of the predominant class with *N* = 38.5.

The VSV helical trunk has three concentric protein layers (Fig. 2c). The innermost layer is the RNA-N protein (RNP) complex, which coils into a left-handed helix with a polarity such that the 3′ end of the (-)-strand genomic RNA is at the tip of the bullet-shaped virions and the 5′ end at the blunt end of the trunk. Two layers of matrix protein (M) surround the RNP, with the same helical symmetry, but with a different packing within each layer. The center of mass (calculated from residues 58–227) of the M2 subunit relates to that of M1 by a 24 Å-radial shift and a 5° rotation around the central axis (with almost no axial shift). A rotation of the shifted M1 by 46° about its center of mass then generates M2. In the early trunk structure, only a single matrix protein layer was placed into the density map, presumably because of the limited resolution of the reconstruction^22^, but a recently published electron cryotomographic (cryo-ET) reconstruction also visualized two M layers^25^. A local resolution estimate for the *N* = 38.5 helical reconstruction (Supplementary Fig. 3d) and analysis of the refined temperature factors in our model (Supplementary Fig. 3e) show that the outer matrix protein (M2) layer is less well defined than the inner matrix protein (M1) layer. We explain this observation by partial occupancy of M2-layer subunits in the outer layer, consistent with the reported stoichiometry of only 1.5 M proteins per N protein in virions^24^. The best-ordered parts of the trunk were the M1 layer and the N-terminal domain (N lobe) of the N protein (Supplementary Fig. 3d, e).

As evident from the superposition of two adjacent asymmetric units on a single common nucleoprotein subunit (Fig. 2d), the molecular contacts that govern coiling of the RNP ribbon into a helix are not fundamentally altered when the number of subunits per helical turn changes from 34.5 to 41.5 between different virions. Our classification analysis suggests a Gaussian distribution with an energy minimum for *N* = 37.5 or 38.5 (Fig. 1c).

**Fig. 3:**
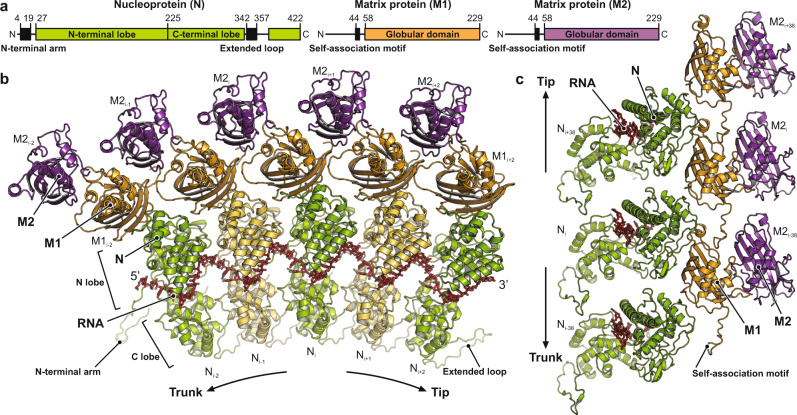
Structure of the RNA and protein subunits within the VSV nucleocapsid. **a** Linear domain organization of the nucleoprotein (N, green) and matrix proteins (M1 and M2, orange and purple, respectively). Amino acid numbers indicate domain boundaries. Globular domains are shown in color. Sequences engaged in inter-subunit contacts are shown as black boxes. **b** Top view of five consecutive asymmetric units along the RNP ribbon. The RNA is shown in red stick representation. Nucleoproteins (N) are colored alternating in green and yellow. The matrix protein of the inner layer (M1) is colored in orange, the one of the outer layer (M2) in purple. Subunits are labeled with indices that increment towards the virion tip, where the 3′ end of the RNA is located, and decrement towards the base of the trunk, where the 5′ end of the RNA is located. **c** Side view of the asymmetric units from three turns of the RNP ribbon. RNA and subunits are colored as in **b**. The tip of the virion would be at the top, the base of the trunk at the bottom. Indices of the individual subunits are shown.

The structures of the N and M subunits in the assembled nucleocapsid determined here (Fig. 3) are generally as observed in previous crystal structures of the individual proteins^3,11–13,19,20,26,27^ (Supplementary Fig. 4a–e). Supplementary Fig. 4f shows per-residue Cα distances after superposition of our N, M1, and M2 structures onto PDB-ID 2GIC (N) and PDB-ID 1LG7 (M), respectively. Most apparent are the different conformations of the N-terminal arm and the extended loop as they pass into contact with the neighboring subunit. While the conformation of residues in those segments that are directly engaged in binding the adjacent subunits are the same (Fig. 3a, b), their linkages are flexible and allow the protein to adopt a more tightly curved arrangement of N in crystallized RNA-N decamers than in the trunk of assembled virions (Supplementary Fig. 4c). We also found conformational differences in two N protein loops (residues 111–133 and 166–181, respectively) (Supplementary Fig. 4c).

The RNA strand lies in a cavity between the N and C lobes of the nucleoprotein (Fig. 3b, c). A different conformation of nucleotide 9, either flipped out or base-stacked on nucleotide 1, was observed in various crystal structures^20^ (Supplementary Fig. 4d) and also when compared to the rabies virus (RABV) RNP structure^28^. In the virion structure here, nucleotide 9 base stacks with nucleotide 1 and is part of a first quasi-helical stretch^3^ together with nucleotides 1–4. The second quasi-helical stretch comprises base-stacked nucleotides 5, 7, and 8. Nucleotide 6 bulges out, separating the two stretches, and its base is not well resolved in our density map. We mapped the N-protein residues that contact the RNA in our structure onto a multiple-sequence alignment that includes sequences from other viruses of the *Rhabdoviridae* family (Supplementary Data 1). Conserved residues _N_Arg143, _N_Lys206, _N_Arg214, _N_Lys286, _N_Arg408, all ordered in our reconstruction with well-resolved side chain density, coordinate the phosphate groups of the RNA backbone by salt bridges. _N_Arg312 probably also contributes a salt bridge, but its side chain is less well resolved. Conserved _N_Tyr152 hydrogen-bonds to the phosphate group of nucleotide 8, and stacks with its aromatic side chain onto the sugar ring of nucleotide 6. The side chains of _N_Arg408 and _N_Arg317 stack on the bases of nucleotides 6 and 4, respectively. Other conserved residues in the binding cleft establish shape complementarity and engage in hydrophobic contacts with the RNA strand. These contacts do not depend on the identity of the base at each nucleotide position and therefore allow the N protein to bind any 9-base sequence in the RNA genome.

### Subunit interactions in the VSV nucleocapsid

To describe the subunit interactions within the helical nucleocapsid, we classified the interfaces according to whether they are within a layer (lateral and axial intra-layer contacts for the N, M1, and M2 layers) or between layers (radial inter-layer contacts). Table 1 summarizes the observed interfaces in the *N* = 38.5 helical structure together with the calculated interface areas (IA), or buried accessible surface areas, for each of the contacts. Interacting residues are labeled in the multiple sequence alignments in Supplementary Data 1–3.

**Table 1:** Subunit interactions in the VSV nucleocapsid

Intra-layer N contacts		IA^a^ (Å^2^)
Lateral intra-layer contacts		
Interface 1	Single stranded RNA bound to N subunit	1055
Interface 2	N_i_ N-terminal arm interacts with N_i-1_ C-terminal lobe and with N_i-2_ extended loop	793, 354
Interface 3	N_i_ extended loop interacts with N_i+1_ C-terminal lobe and with N_i+2_ N-terminal arm	683, 354
Interface 4	N_i_ N-terminal lobe interacts with N_i+1_ N-terminal lobe	442
Interface 5	N_i_ C-terminal lobe interacts with N_i+1_ C-terminal lobe	207
Axial intra-layer contacts		
Interface A	N_i_ subunit interacts with N_i+39_ subunit	94
Interface B	N_i_ subunit interacts with N_i+38_ subunit	18

Intra-layer M1 contacts		IA (Å^2^)
Lateral intra-layer contacts		
Interface 6	M1_i_ globular domain interacts with M1_i+1_ globular domain	65
Axial intra-layer contacts		
Interface C	M1_i_ self-association motif interacts with M1_i-38_ globular domain	400
Interface D	M1_i_ globular domain interacts with M1_i+39_ globular domain	78

Intra-layer M2 contacts		IA (Å^2^)
Axial intra-layer contacts		
Interface E	M2_i_ globular domain interacts with M2_i+38_ globular domain	49

Inter-layer N-M1 contacts		IA (Å^2^)
Radial inter-layer contacts		
Interface i	N_i_ C-terminal lobe interacts with M1_i_ globular domain	345
Interface ii	N_i_ C-terminal lobe interacts with M1_i-38_ globular domain	310
Interface iii	N_i_ C-terminal lobe interacts with M1_i+1_ globular domain	117

Inter-layer M1-M2 contacts		IA (Å^2^)
Radial inter-layer contacts		
Interface iv	M1_i_ globular domain interacts with M2_i_ globular domain	600
Interface v	M1_i_ globular domain interacts with M2_i+1_ globular domain	430

^a^IA: interface area (buried accessible surface area upon interface formation).

#### Intra-layer contacts

**Fig. 4:**
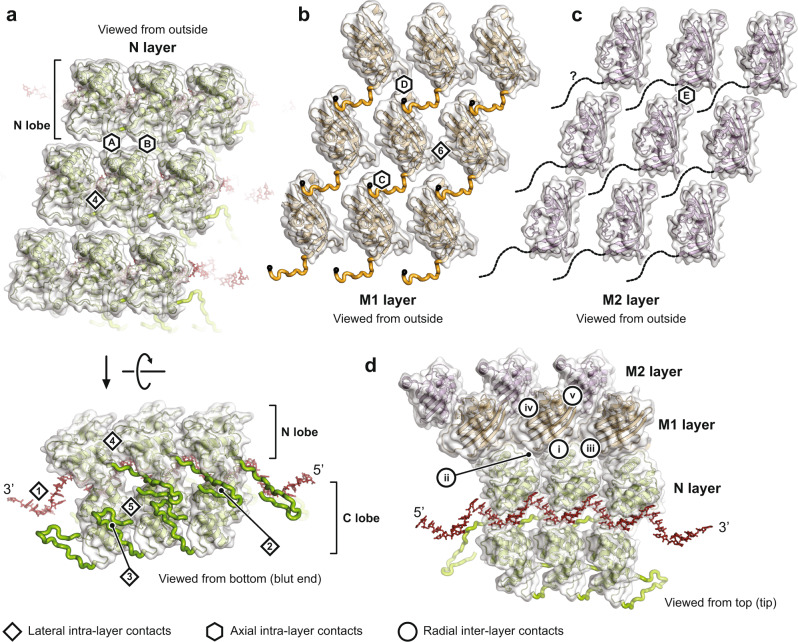
Inter-subunit contacts. RNA is shown in red stick representation. Globular domains are shown with transparent surface, extensions as thick ribbon. Lateral intra-layer contacts are labeled with diamonds and Arabic numerals, axial intra-layer contacts with hexagons and letters, and radial inter-layer contacts with circles and Roman numerals. See also Table 1. **a** Inter-subunit contacts within the N protein layer. Top, side view of the N layer from the outside. Bottom, bottom view of the N layer. **b** Inter-subunit contacts within the M1 protein layer in side view from the outside. **c** Inter-subunit contacts within the M2 protein layer in side view from the outside. **d** Inter-subunit contacts between the N, M1, and M2 layers.

The N protein clamps onto the RNA strand (interface 1) and forms extensive contacts with adjacent N subunits thought its N-terminal arm and extended loop (Fig. 4a, bottom). The segments of these extensions that bind the C lobes of the preceding (interface 2) and following (interface 3) N subunits, respectively, are essentially as observed in the crystal structures. The N-terminal arm of an N subunit folds through a groove in the subunit 5′ to it along the RNA; the extended loop folds against the blunt-end facing surface of the subunit 3′ to it and also interacts with the N-terminal arm of the next N subunit in the 3′ direction. These elaborate imbrications would tie together the N-protein ribbon even without the continuity of the RNA polynucleotide chain, and we have suggested elsewhere that they indeed do so when the RNA passes into the polymerase active site, retaining continuity of the RNP upstream and downstream of the “transcription bubble”. The tethers are flexible as they pass from their subunit of origin into the neighboring subunit, allowing variability of overall radius of the RNP helix, as seen in the widening turns at the tip and in the distribution of trunk diameters, while maintaining a constant 9-nucleotide spacing along the RNA strand. When the RNP complex coils into the helical nucleocapsid, there are additional lateral contacts between N-protein neighbors (Fig. 4a, bottom). Between adjacent N-lobes, salt-bridges form between _N_Glu169 and _N_Arg179 and between _N_Asp85 and _N_Lys34 (interface 4). C-lobe contacts (interface 5), closer to the helix axis, are much less extensive. Axial contacts in the N layer (interfaces A and B) are likewise tenuous, where N_i_ fits loosely into the shallow groove between N_i+38_ and N_i+39_ (Fig. 4a, top).

In the only substantial intra-layer contact in the M layers, the “self-association motif” (residues 44–52)^27^ creates a chain of interactions within the M1 layer, and possibly within the M2 layer (Figs. 3a, c and 4b, c). The motif from M1 subunit i interacts with a pocket defined by residues 78–84 and 114–125 in M1 subunit i + 38 (interface C), as predicted from lattice contacts in crystals of VSV M^27^. Poor M2-layer resolution prevented us from determining whether the same axial contact is also present there; the positions and orientations of M2 subunits would allow the contact to be present. Other intra-layer contacts (interfaces 6, D and E) are very slight and unlikely to contribute to overall stability (Table 1).

#### Inter-layer contacts

Each N protein contacts three M1 subunits (Fig. 4d and Table 1). The first of these radial inter-layer contacts, interface i, is the most extended, and the one that is also present in the tip of the VSV bullet (see below). The contact is well resolved in our local reconstruction and involves N protein residues 116–125 from a long loop that wraps around the N lobe domain and binds two M1 subunit loops, residues 94–101 and 149–152. The M1 residues of this interface are conserved (Supplementary Fig. 5 and Supplementary Data 2). Stabilizing interactions include hydrogen bonds between the main chain of the N loop 116–125 with the M1 loop 94–101. We also observe hydrophobic contacts between residues _N_Val121 and _M1_Met98 and _N_Val116-_N_Leu117-_N_Pro118 and _M1_Pro149-_M1_Pro150. The second contact of N with an M subunit, interface ii, is with M1_i-38_. Salt bridges are formed between _N_Lys78 and _M1_Glu130, and between _N_Asp100 and _M1_Arg159. Most other residue contacts in this interface are also polar. The third, minor contact, interface iii, is between the N protein C-terminal lobe and the M1_i+1_ globular domain.

Each M1 protein contacts two M2 subunits (Fig. 4d and Table 1). Interface iv is an extended contact with the M2_i_ subunit. _M1_Asp191 binds _M2_Arg102. The M2 C terminus with its _M2_Phe228 inserts into a hydrophobic cleft of M1 where it interacts with _M1_Trp220. Additional hydrophobic contacts are between _M1_Pro187 and _M2_Met94-_M2_Ile96. Interface v is a contact with the M2_i+1_ subunit.

The buried accessible surface areas for each interface (Table 1) and residue conservation analysis (Supplementary Fig. 5) support the notion that the VSV nucleocapsid is held together primarily by lateral interactions in the N layer (interfaces 1–3), axial interactions in the M1 layer (interface C), and radial interactions between the layers (interfaces i and iv). Indeed, surface residues of those interfaces generally show the highest conservation, and deletion of resides in the N-terminal arm or extended loop both result in loss of N oligomerization^19^. Proteolytic cleavage of the M protein likewise prevents self-association in vitro^11^.

### Structure of the VSV tip

**Fig. 5:**
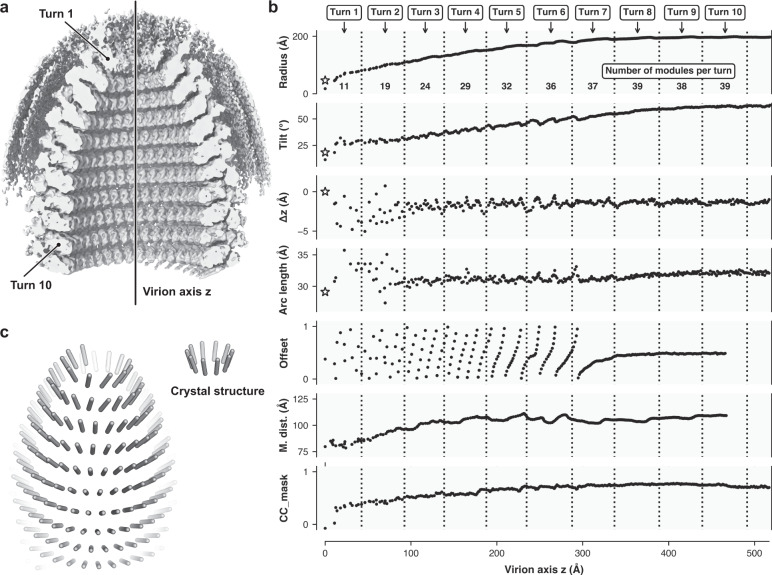
Structure of the VSV tip. **a** Asymmetric reconstruction of the VSV tip. The density was low pass filtered at 8.8 Å resolution and sharpened by applying a *B* factor of −100 Å^2^. To allow inside view, the density is partially cut. **b** Analysis of individual modules after rigid body fitting into the map of the VSV tip reconstruction. The horizonal axis is the distance from the top of the tip along the virion axis. Each dot corresponds to one module. Turns are delineated on top of the plot. Asterisks in the plots are values calculated from the crystal structure of the decameric N-RNA complex^3^. Radius: distance between the virion axis and the center of gravity of the nine RNA bases. Tilt: tilt of the N protein relative to the helical axis. Δz: position of the module along the virion axis (RNA center of gravity). Arc length: lateral distance between adjacent modules (RNA center of gravity). Offset: Relative offset of the module with respect to the position of the modules in next turn (dihedral angels, RNA center of gravity). M. dist.: distance between the module (RNA center of gravity) and the middle of the lipid bilayer. CC_mask: correlation coefficient between the rigid-body fitted module (N and M1 subunits) and the map of the tip reconstruction. **c** Schematic representation of the N subunit arrangement in the VSV tip. The longitudinal axis of each N subunit is shown (left) and compared to the N arrangement in the decameric crystal structure (right).

With a stepwise particle alignment and reconstruction protocol, which we describe in detail in the Methods section and Supplementary Fig. 6, we obtained an asymmetric reconstruction of the rounded VSV tip (Fig. 5a). The reconstruction comprising the first ten turns of the RNP ribbon had a nominal resolution of about 9 Å and allowed a rigid body-fit of the structures of individual modules (Supplementary Fig. 7). The density along the RNP ribbon showed that binding of M1 to N (interface i) within each module in the tip is identical to the corresponding interaction in the modules of the trunk. The same appears to be true for binding of M2 to M1 (interface iv), but partial occupancy of the M2 subunit resulted in weak density for the M2 layer generally.

The first turn of the RNP ribbon at the tip of the virus, where the 3’ end of the genomic RNA is located, comprises about 11 modules. The number of modules then increases in each subsequent turn, up to turn eight, where we counted 39 modules, which corresponds to the number of modules per turn in the regular helical structure of the nucleocapsid trunk (Fig. 5b). A plot of the radial distance between the nine RNA bases (center of gravity) of each fitted module and the virion axis shows that the radii increase gradually from one module to the next (Fig. 5b). Concomitant with this radial expansion, the tilt of modules steadily increases for an accumulated change of about 40° (Fig. 5c). The relative shift along the virion axis and the arc length between adjacent N subunits are both approximately constant. The constant lateral distance between neighboring N proteins allows the 9 nucleotide-register of RNA binding (interface 1) as well as interaction of the N-terminal arm (interface 2) and the extended loop (interface 3) to be maintained, independent of the curvature of the turns. The axial interfaces between subunits that we observed after the tip-to-trunk transition (Table 1) are, however, not conserved in coils of the tip. The difference is evident from the offset plot (Fig. 5b). In the trunk the offset is constant at 0.5, meaning that each N subunit sits exactly between two N subunits of the next turn. In the tip, however, the offset oscillates between 0.0 and 1.0, even within a single turn. The RNP ribbon in the tip essentially “slides” on the preceding turns without establishing invariant axial binding interfaces. We also measured the distance between the inner membrane leaflet and the nucleocapsid, which shows that the spacing is approximately 20 Å shorter at the tip than in the trunk (Fig. 5b).

### Binding of the phosphoprotein P to the nucleocapsid

**Fig. 6:**
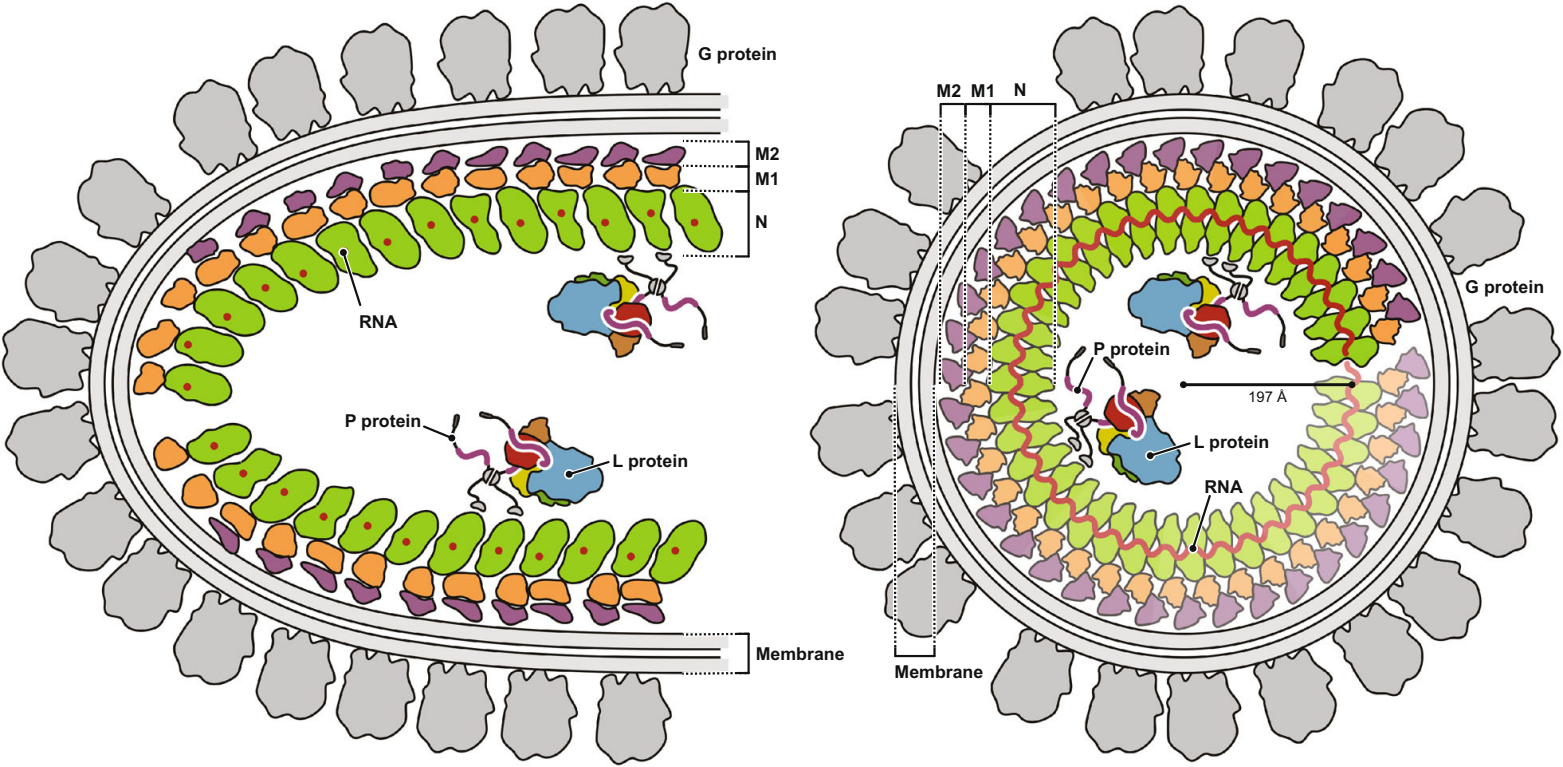
Model of VSV. Schematic diagram of VSV. A longitudinal section of the VSV tip is shown on the left, a cross section of the helical trunk on the right. All viral proteins are drawn to approximate scale.

Mature VSV virions contain approximately 50 copies of the L protein^24^, which are incorporated into virions by its phosphoprotein (P) cofactor (Supplementary Fig. 8a). From published work, we know the structures in isolation of the P_L_ segment bound to the L protein^15^ and the P_CTD_ bound to the N protein^12^. We found density of the P_CTD_ domain in situ, bound to the N protein in the interior of the virus, when we low-pass filtered and low-level contoured maps of our trunk reconstructions (Supplementary Fig. 8b). Attempts to classify sub-particles for modules with bound P_CTD_ were not successful, likely because of the size of the P_CTD_ domain (8 kDa) and the relatively low occupancy. Nonetheless, the observation of P_CTD_ at low resolution in a conformation essentially as in the crystal structure^12^ and in a recent tomographic reconstruction, in which P_CTD_ appears at the same position^25^, further supports the current model of how P tethers L to the RNP for packaging into the virion (Fig. 6).

### Membrane association of M and G

The N-terminal extensions of M subunits in both layers project toward the membrane (Supplementary Fig. 8c, d). A segment containing eight lysines (“basic N terminus”, residues 5–19) crosslinks efficiently to photoactivatable membrane probes in VSV^29^, and 24 residues then intervene between the C-terminus of this cross-linkable segment and the N-terminus of the ordered self-association motif (residue 44). In our reconstruction of the helical trunk, a gap of about 30 Å separates the M2 layer of the nucleocapsid from the inward-facing headgroups of the viral membrane (Supplementary Fig. 8d). If the M2-layer self-association motif were to dock to an axially shifted subunit, as in layer M1, 24 residues could readily bridge the gap between residue 44 and the bilayer, and even the corresponding segment from, M1 could do so, as the distance (approximately 50 Å) is only slightly greater than 2 Å per residue. We do not see ordered density for the G protein, consistent with biochemical evidence that the cytoplasmic tail of G does not interact strongly with the matrix protein^30^ and with observations that infectious chimeric viruses can be engineered in which other viral fusion proteins substitute for VSV G^31^.

## Discussion

High-resolution cryo-EM reconstructions depend on averaging images of a set of homogenous particles (in composition and or conformation) after determining their projection angles and shifts. Classification of heterogenous images can be particularly challenging if the individual particles have a high degree of local symmetry (tip structure here) or have different, but related strict helical symmetries (trunk structures here). Our supervised classification protocol partitioned the virus images into classes with different helical symmetries (number of subunits per turn), and subsequent locally focused alignment of sub-particles yielded a reconstruction of the basic trunk-building module at 3.5 Å resolution (Fig. 1). For the tip reconstruction (Fig. 5) we used a stepwise alignment protocol (Supplementary Fig. 6). The barely sub-nm resolution suggests that there is considerable heterogeneity among individual tip structures; cryo-ET methods might be required to understand their structural variation.

The overall resolution of the reconstruction we have obtained, and the even higher resolution of an N-RNA-M1-M2 module, has allowed us to integrate published observations into a likely sequence of events during VSV budding at the plasma membrane of an infected cell. As also observed previously^22^, comparison of the initial turn of the RNP helix with the crystal structure of an RNP decamer^3^ shows that the lateral interactions and tilt of the subunits with respect to the axis are very similar (Fig. 5c). Recombinant rabies virus N forms a closely related undecameric ring^28^. When expressed in insect cells, N binds longer RNA molecules and forms undulating ribbons with varying local curvature^23^. At very low ionic strength and neutral pH, these ribbons condense locally into strings of unidirectional, tip-like conical structures that expand in 5 turns from a tight ring at their apex to a diameter of about 390 Å, and at pH 5, the RNP forms bullet-shaped structures very similar to the RNP in virions, but with a somewhat narrower diameter, corresponding to 32–34 subunits per turn^23^. These observations show that N alone can determine the size and shape of the particle and that M, which appears to modulate slightly the most stable diameter for a continuous helical assembly in the trunk of a particle, couples assembly of the RNP bullet to the membrane though which it buds.

Comparing the structure of the first 8 turns of the RNP helix at the tip of the virion with published structures of N and RNPs in various forms as just described, we can propose the following picture for VSV assembly at the membrane. Budding begins at the domed end, with the 3’-end of the RNP. The similarity of the crystallized RNA ring and the first turn of the RNP in virions suggests that the ring is a good approximation to the structure that initiates virion assembly. When bound on an RNA molecule longer than 90 nucleotides, a decameric ring cannot close on itself and must instead dislocate into a lockwasher and propagate as a helix. Stacking of N subunits in successive turns, even if relatively non-specific, and the steep tilt of the subunits will then cause the second turn to have a larger diameter than the first, and similarly in increasingly wide gyres until a stable axial stacking occurs after about 8 turns, with 38.5 N subunits per turn. The energy required to curve the membrane will also favor successively greater diameters as the tip propagates, until a balance between membrane deformation and stable, half-offset axial packing occurs at the tip-to-trunk transition.

The orientation with which N binds RNA requires that the 3’ end of the genome be at the domed end, with an exposed extended loop of the C lobe of the first N protein. Initiation of assembly at the membrane might simply be a linkage through M of the subunits in the first, 10–11 subunit turn of the RNP with the inner leaflet of the membrane lipid bilayer. Alternatively, a specialized organization of the 3’-end of the RNP might create a more specific initiation complex. The resolution of the tip structure at that position does not allow a firm conclusion. One speculative possibility is that the RNA 3’ end associates with the template entry channel of a molecule of the viral RdRp L protein, just as it does to impart specificity of viral RNA plus-strand packaging in the dsRNA viruses, with one polymerase per genome segment^32–34^. Such an arrangement could be coupled to M-protein suppression of L catalyzed polymerization^35^. Influenza virus assembly also includes a 3’-end localized RdRp molecule on each of the eight genomic RNAs^36^.

Detecting and visualizing the M2 layer and building a well-positioned model for both M1 and M2 are important consequences of the resolution of our reconstruction. Previous work had assumed that the “extra” M (present in total about 1.5 times the level of N) decorated the inner surface of the helical RNP assembly, as suggested in the “cigar” model of virus assembly in which the N-RNA wraps around a core of M^37^. The presence of two layers of M around the N-RNA now provides an explanation for the remaining M in the virion. A key interaction between successive turns of the helical ramp is docking of the M1-layer self-association motif from subunit i into the pocket that receives it on subunit i + 38. Although the short segment connecting the motif itself with the globular region of M may be flexible, a loop from subunits in the M2 layer bears against it, potentially reinforcing its conformation and helping to establishing the half-staggered offset of successive turns. In the tip, where the offset varies, the M2 layer appears to be absent.

The M2 layer is only half occupied. Our current model does not show whether its distribution is systematic or random. The self-association motif could establish some degree of longitudinal coherence, so that variable-length strips of motif-linked, M2-layer subunits would decorate the outside of the M1 layer. A cryo-ET structure of the RABV trunk shows only a single M layer^38^, consistent with an approximately one-to-one ratio of the number of N and M molecules^39^, and further studies should clarify functional roles of variations in the nucleocapsid organization among virions of the *Rhabdoviridae* family. *Note added at proof* Zhou et al. have obtained similar results for the RNP-M-protein trunk^40^.

## Methods

### Virus production and purification

VSV Indiana serotype was grown on BSR-T7 cells seeded in DMEM (Corning, cat. # 10-013-CV) − 10% FBS (Tissue Culture Biologicals, cat. # 101), and infected one day later at a multiplicity of infection (MOI) of 3 for 1 h. The virus suspension was then replaced by DMEM − 2% FBS, and cell supernatant was harvested 20 h post-infection. The supernatant was first clarified by centrifugation at 750 × *g* for 5 min, and concentrated through a 15% (w/v) sucrose (Sigma, cat. # S9378-10KG) cushion in NTE buffer (10 mM Tris-HCl pH 7.4 [Fisher Scientific, cat. # T395-1], 100 mM NaCl [Millipore, cat. # SX0420-3], 1 mM EDTA [MACRON Fine Chemicals, cat. # 4931-04]) at 110,000 × *g* for 2 h at 4 °C. Pellets were resuspended overnight at 4 °C in NTE buffer, put on top of a linear 15–45% (w/v) sucrose gradient prepared in NTE buffer, and centrifuged at 200,000 × *g* for 3 h at 4 °C. The band corresponding to virus was collected by side-puncture of the tube in a volume of 0.5 mL, and dialyzed overnight at 4 °C against 1 L of NTE buffer using a Slide-a-Lyzer (ThermoFisher Scientific, cat. # 66454), 0.1–0.5 mL, 10 kDa cut off, cassette.

### Specimen preparation and cryo-EM data collection

VSV particles were immobilized in vitreous ice using a CP3 cryoplunger. 3 μl of band-purified virus was applied to copper mesh holey carbon grids (C-flat 1.2 μm diameter holes with 1.3 μm spacing and a support thickness of ~40 nm) and blotted for 3 s in a chamber maintained at 90% humidity before plunge-freezing into liquid ethane at −171 °C. Images of VSV particles were collected on a Titan Krios G3i microscope (Thermo Fisher Scientific) operated at 300 kV, and equipped with a pre-camera energy filter (Gatan Image Filter) and K3 Summit direct detector (Gatan). The nominal magnification was 105 kx, corresponding to a calibrated physical pixel size of 0.85 Å. Dose-fractionated movies were recorded in counting mode with 0.05 s per frame for a total of 2.5 s (50 frames, total dose of 60 electrons per Å^2^). Using SerialEM (v3.7.1–64 bit)^41^, we recorded a total of 27 movies for each stage position (nanoprobe mode with an illuminated area of ~1 μm, 3 exposures per hole from a total of 9 holes).

### Cryo-EM data processing

#### Particle picking and movie processing

Movie frames were aligned and summed using MotionCor2 (5 × 5 patch alignment)^42^. In four times binned and low-pass filtered sums (18,353 micrographs) we manually marked 12,348 VSV trunks with e2helixboxer.py from EMAN2^43^. We next estimated magnification distortion from power spectra of trunks extracted from the original images, as previously described^44^, applied the fitted values (distortion angle = 109.96, major scale = 1.0026, minor scale = 0.9974) with mag_distortion_correct^45^ to the original movie stacks, and used again MotionCor2 through the RELION^46^ wrapper relion_run_motioncorr to obtain sums from the magnification corrected movie stacks.

#### Particle extraction and CTF determination

Determination of contrast transfer function (CTF) parameters, extraction of helical trunk segments, and particle stack preparation involved the following steps: (i) Manually picked trunk coordinates from EMAN2 were converted to RELION star file format. (ii) Using relion_preprocess, we extracted helical segments based on the trunk coordinates (box size = 1200 × 1200 pixels, helical_nr_asu = 37.5, helical_rise = 1.38 Å). Background radius was set to 482 pixels and the helical outer diameter to 820 Å. Segment images were normalized and contrast-inverted. (iii) We used Gctf^47^ to determine CTF parameters from the total-summed images with refinement of values at individual segment coordinates. (iv) Unbinned and binned particles stacks were created from the extracted segment images. (v) We selected 163,855 segments (95%) from the initial particle stack by one round of 2D classification with relion_refine (using 2-times binned data), discarding mostly segments that were extracted too close to the base or tip of the VSV bullet.

#### Initial 3D reconstruction

We obtained an initial 3D reconstruction from a subset of 871 segments selected from a visually good-looking 2D class. Using relion_reconstruct we prepared a first 3D reference by just applying the alignment parameter from the 2D alignment and C_1_ symmetry (using 4-times binned data). With auto-refinement in relion_refine (helical_inner_diameter = 260 Å, helical_outer_diameter = 700 Å, helical_nr_asu = 35, helical_z_percentage = 0.1, helical_twist_initial = −9.6°, helical_rise_initial = 1.379 Å) and imposing the previously reported helical symmetry (37.5 subunits per turn, helical pitch = 51.7 Å)^22^, we obtained a 8.9 Å resolution reconstruction.

#### Helical reconstruction protocol

We set up an iterative helical reconstruction scheme using programs from Frealign^48^, cisTEM^49^, RELION^46^, and EMAN2^43^ where each cycle involved the following steps: (i) References-based alignment of particle segments with refine3d (version 1.01) (first cycle in mode 3, subsequent cycles mode 1, C_1_ symmetry). (ii) 3D reconstruction of the full and half maps based on the alignment parameters with frealign_v9.exe (version 9.11) (C_1_ symmetry). Note that for reconstruction of the half maps, we used the first and second half of the particle segments (and not even and odd numbers, respectively) in order to prevent inflation of the half-map Fourier shell correlation (FSC) that would have resulted if segments extracted from the same trunk ended up contributing to different half maps. We also turned off Wiener filtering (FFILT = F) in order to avoid artifacts in the reconstructions, presumably because of the non-standard spectral signal-to-noise ratio of helical structures (e.g. layer planes in reciprocal space with very strong signal and no signal in between). (iii) Helical symmetry refinement with relion_helix_toolbox with the C_1_-symmetric map from the previous step as input (cyl_inner_diameter = 246–326 Å [depending on the number of subunits per turn], cyl_outer_diameter = 546–626 Å [depending on the number of subunits per turn], z_percentage = 0.3, sphere_percentage = 0.9). Symmetry search was local with a search range of 0.95–1.05 times the initial value for the helical rise, and of a helical twist corresponding to ±0.1 of the initial value of the number of subunits per turn. (iv) Real space helical symmetrization of the full and half maps with relion_helix_toolbox (z_percentage = 0.3, sphere_percentage = 0.9). (v) Masking of the helically-symmetrized maps with a cylindrical shell mask (inner radius = 123–163 Å, outer radius = 273–313 Å) with e2proc3d.py. (vi) FSC calculation from the half maps with e2proc3d.py. Applying this reconstruction protocol with the previously reported helical symmetry^22^ to all 163,855 segment particles resulted in a map of about 7.5 Å resolution (using 2-times binned data).

#### Symmetry and geometry analysis

To determine the apparent rotational symmetry from top views of partially assembled or disrupted nucleocapsids (Supplementary Fig. 1b, left), we calculated for each image the rotational self-correlation within a radius of 153–255 Å with “OR MAP” from SPIDER^50^ (Supplementary Fig. 1b, middle, blue curve). We numerically fit *CC* of Eq. (1) to the observed self-correlation within 5–60° of the self-rotation angle ϕ using SciPy^51^ (Supplementary Fig. 1b, right, red curve).

1 $$CC=a \,{{{{{\rm{cos }}}}}}\left(b\,\phi+c\right)+{fpln}(\phi )$$

2 $$N=b\frac{{360}^{\circ }}{2\pi }$$

A third degree polynomial *fpln* was first obtained from an initial fit (Supplementary Fig. 1b, middle, green curve), and the number of submits per turn *N*, or apparent rotational symmetry, is given by Eq. (2).

To measure the width and the length of VSV bullet particles, we extracted 2-times binned images (1400 × 1400 pixels) centered at the middle of each manually picked trunk. We used relion_autopick as a bilayer-enhancement algorithm by calculating a figure-of-merit image for each VSV bullet image based on a membrane reference image as input. For each particle that was fully within the extracted box, we manually measured its length, with e2helixboxer.py, by defining points centered on the membrane at the apex and bottom, respectively. To measure its width, we first projected density of the membrane-enhanced images along the particle axis, and then used Python to locate the two membrane peaks within the 1D-density distribution.

#### Supervised classification of helical segments

To prepare 3D references with different geometries for supervised classification of helical segments, we expanded the asymmetric unit density of the initial 7.5 Å-resolution reconstruction. We chose to describe flattening due to specimen preparation by modeling assemblies as elliptical helices. For this, we first rigid-body fitted into the 7.5 Å-resolution reconstruction density one asymmetric unit consisting of one N protein subunit with 9 RNA nucleotides from PDB-ID 2GIC^3^ and two M protein submits from PDB-ID 1LG7^11^, and then used the fitted models to generate a mask for density extraction. We defined a reference point within the extracted density, and a reference vector chosen normal to the tangential plane of the helix. Expansion then involved repetitive placement of the reference point for the asymmetric-unit density along the path of an elliptical helix while keeping the reference vector normal to the tangential plane of the elliptical helix (Fig. 1b). The geometry of the resulting 3D references is defined by the geometry the elliptical helix and the number of subunits per helical turn. The pitch of the elliptical helix *P* is related to the helical rise *Δz* and the number of subunits per turn *N*:

3 $$P=\triangle {z\; N}$$

The major (long) and minor (short) axis of the elliptical helix, *a* and *b*, depend on the number of subunits per turn *N*, the distance along the helical path between reference points of adjacent asymmetric subunits (where *d*_*xy*_ is its projection into the *xy*-plane), and the degree of degree of ellipticity (or flattening) *f*:

4 $${U}_{{{{{{\rm{circle}}}}}}}=2\pi \,r={d}_{{xy}}\,N$$

5 $$a={r\; f}$$

6 $$b={{{{{\rm{function}}}}}}\left({U}_{{{{{{\rm{ellipse}}}}}}},a\right)\,{{{{{\rm{where}}}}}}\,{U}_{{{{{{\rm{ellipse}}}}}}}={U}_{{{{{{\rm{circle}}}}}}}$$

We used SciPy^51^ for calculation of elliptical line integrals and to numerically determine *b* from the elliptical circumference *U*_ellipse_ and *a*. We also used the Biopython module Bio.PDB for coordinate manipulations within Python^52^. In summary, the values of *P*, *N*, *d*_*xy*_, and *f* define each 3D reference.

We first used our subset of 871 segment particles and aligned them with refine3d (mode 3) to a set of non-flattened (*f* = 1.00) 3D references with constant pitch *P*, different numbers of subunits per turn (*N* = 34.5, 35.5, 36.5, 37.5, 38.5, 39.5, 40.5, and 41.5), and a varying *d*_*xy*_. The pitch *P*, which we obtained by Fourier analysis of segments of trunk images, was kept constant, because it is determined by the axial stacking of helical rungs and does not depend on *N* or *f*. Class assignment (where each class corresponds to one particular 3D reference) was based on the highest alignment score and resembled a Gaussian distribution with respect to *N* (Fig. 1c). For each *N*, distributions peaked sharply at a same value for *d*_*xy*_. A constant distance between adjacent subunits would be expected if the number of RNA nucleotides per N protein were constant among assemblies with different numbers of subunits per helical turn. We therefore kept the same value for *d*_*xy*_ during subsequent analysis.

For classification of the full segment particle stack with 163,855 images, we relied on a high performance computer cluster that allowed us to run parallel alignment calculations on up to 2000 CPUs. Using 4-times binned data, we first globally aligned all images to eight non-flattened 3D references (*N* = 34.5, 35.5, 36.5, 37.5, 38.5, 39.5, 40.5, and 41.5) using refine3d (mode 3). For each of the eight global alignments, we expanded the particle stack 39-fold according to the helical symmetry given by *N* using relion_particle_symmetry_expand. The expanded particle stack (6,390,345 images) was then locally aligned to 96 3D references using refine3d (mode 1), where we provided for each number of subunits per turn (*N* = 34.5, 35.5, 36.5, 37.5, 38.5, 39.5, 40.5, and 41.5) twelve 3D references with different degrees of flattening (*f* = 1.000, 1.004, 1.007, 1.011, 1.015, 1.018, 1.022, 1.025, 1.029, 1.033, 1.036, and 1.040). Class assignment and particle selection was again based on the highest alignment score with respect to the 3D references and expanded images, respectively (Fig. 1c).

#### Helical reconstructions

We selected segment particles from the different *N*-classes without flattening and with only minimal fattening and applied for each particle stack a helical reconstruction protocol as outlined above using 2-times binned data. The number of helical segment particles ranged from 228 (*N* = 41.5) to 21,530 (*N* = 38.5) (Supplementary Table 1). Helical reconstructions were then calculated with non-binned images and used for particle polishing with relion_motion_refine. After another round of the helical refinement protocol using the polished particle stacks, the resolution of the helical reconstructions, as determined by half-map FSC after masking, ranged from 12.1 Å (*N* = 41.5) to 4.1 Å (*N* = 38.5) (Supplementary Table 1 and Supplementary Fig. 3a). Beam tilt correction did not further improve the resolution of the reconstructions. Local resolution was estimated with the program ResMap^53^. We post-processed the *N* = 38.5 map by first sharpening it with cisTEM’s sharpen_map^49^. The sharpened map was then used as input for LocScale^54^, together with a preliminary refined model with estimated B factors. The resulting locally contrast-optimized map was again sharpened with sharpen_map.

#### Local reconstruction

To calculate a local reconstruction from the average of all asymmetric subunits (comprising one N protein, nine RNA bases, and two M proteins) captured in helical segments, we extracted subparticles as previously described^32,55^. For each *N* class, subparticles with a box size of 256 × 256 pixels were extracted with IMOD (v4.9.10)^56^ around the central helical turn, because segment images were initially extracted with a one-turn offset. Subparticle stacks were signal-subtracted using relion_project and a mask that excluded the asymmetric subunit. The signal-subtracted stacks from the different *N*-classes were aligned with respect to each other based on transformation matrices determined from rigid-body fitted PDB models. The resulting subparticle stack, containing approximately three million images, was further aligned by classification without alignment using three iterations of the following protocol: classification with cisTEM’s refine3d (8 classes, 5 Å high-resolution limit for classification, 60 cycles, 3D mask covering the asymmetric unit), superposition of the maps corresponding to the 8 classes based on rigid-body fitted models, and updating of the particle alignment parameters according to the 3D map alignment. As a final step, we carried out local alignment with refine3d (5 Å high-resolution limit for refinement, 8 cycles, 3D mask covering the asymmetric unit). The masked local reconstruction of the asymmetric unit, calculated from a non-signal-subtracted particle stack, had a nominal resolution of 3.5 Å (Supplementary Table 1 and Fig. 3b). We post-processed the map by sharpening it with sharpen_map^49^ and local filtering with LocScale^54^.

#### VSV tip reconstruction

To obtain a reconstruction of the VSV bullet tip, we initially selected 5763 projection views of viral particles from the micrographs, in which we manually marked the base and tip of each bullet (Supplementary Fig. 6a). A segment extracted at the center of each particle was used to determine *N*, the number of subunits per turn in the helical region of the stalk, by supervised classification as described above (without taking flattening into account), yielding 2521 particles with *N* of 38.5 (Supplementary Fig. 6b). The alignment determined at the center of the particles was then “shifted” towards the tips, to the point before the helical part transitions into narrowing turns. We did this stepwise according to helical symmetry with increments of ten, five and finally one asymmetric unit shifts, until the distance between the projection of a reference point on the z axis within the box of the 3D reference and the projection of the manually marked tip on the projection of the *z-*axis in each particle image was minimal (Supplementary Fig. 6c and Supplementary Movie 1). At each shift, we updated the alignment by local refinement with cisTEM’s refine3d. We then further aligned the tip images using classification without alignment (3 cycles 2D classification, and 1 cycle 3D classification). For this, we calculated translational offsets along the *z*-axis between the individual classes from rotational averages and applied corresponding shifts according to helical symmetry (Supplementary Fig. 6d, e). Next, we symmetry expanded each tip image (+/−80 asymmetric subunits along the helical symmetry) and calculated scores using a 3D reference with a rotationally averaged membrane region, reconstructed without the particle image for which the score was calculated. (Supplementary Fig. 6f, helical offset alignment cycle). This helical offset alignment was iterated for about 9 cycles until convergence (Supplementary Fig. 6g). As the reconstruction improved, we rigid-body fitted the structures of individual modules (N and M1 subunits) where the density was good enough to warrant an unambiguous fit. The fitted modules where then used to calculate a mask and transformation matrices for local symmetry density averaging. The resulting reconstruction was then used for particle picking in the original micrographs using cisTEM’s match_template, refine_template, and make_template_result^57^ (Supplementary Fig. 6h), yielding a stack of 25,401 images, which were then aligned with cisTEM (mode 3, global alignment, C_1_ symmetry) (Supplementary Fig. 6f, big cycle with 2D template matching).

### Model building and refinement

Templates for modeling the N protein were taken from PDB-IDs 2GIC^3^ and 5UK4^26^. The M proteins where modeled based on PDB-ID 1LG7^11^. We used the programs O^58^ for model building and adjustments, phenix.real_space_refine^59^ for structure refinement (standard stereochemical and *B*-factor restraints, as well as Ramachandran, rotamer, and secondary structure restraints), phenix.mtriage for FSC calculations between maps and models^60^, and MolProbity^61^ to validate the models (Supplementary Table 1).

A model that was refined against the post-processed local reconstruction map had the following composition, where the protomer indices refer to the numbering in the *N* = 38.5 helix: 13 RNA nucleotides modeled as poly-uridine (2 from N_i−1_, 9 from N_i_, and 2 from N_i+1_); nucleoprotein (N) residues 2–35 (N-terminal arm from N_i+1_), 20–341 and 366–422 (N- and C-terminal lobes from N_i_), 335–373 (extended loop from N_i−__1_); matrix protein (M1) residues 43–59 (self-association motif from M1_i+38_), 57–227 (globular domain from M1_i_); matrix protein (M2) residues 58–229 (globular domain from M2_i_). Compared to the N protein structures of PDB-IDs 2GIC and 5UK4, we corrected a sequence register shift at residues 154–181. The FSC between the refined model and final local reconstruction map was 0.5 at a spatial frequency corresponding to a resolution of 3.9 Å (Supplementary Fig. 3b), suggesting that the 3.5 Å nominal resolution determined from the half maps was slightly overestimated.

We also fit the model from the local reconstruction into the 4.1 A-resolution map of the *N* = 38.5 reconstruction. For refinement with phenix.real_space_refine, we placed 15 copies of the asymmetric unit by stacking three partial turns with five consecutive units each. This allowed the central asymmetric unit to form all potential inter-subunit contacts during refinement. The asymmetric unit of the refined helical structure had the following composition: 9 RNA nucleotides modeled as poly-uridine; nucleoprotein (N) residues 2–422; matrix protein (M1) residues 43–227; matrix protein (M2) residues 58–229. The FSC between the refined model and final helical reconstruction map with *N* = 38.5 was 0.5 at a spatial frequency corresponding to a resolution of 4.6 Å (Supplementary Fig. 3c).

The refined structure of the asymmetric unit from *N* = 38.5 was then fit into the maps of the other *N* classes by rigid-body fitting (Fig. 2a). Each subunit was treated as a rigid-body, except in case of the low-resolution map from *N* = 41.5, for which the entire asymmetric unit was defined as a rigid body.

### Figure preparation

We prepared the figures using PyMol (The PyMOL Molecular Graphics System, Version 2.1 Schrödinger, LLC) and matplotlib^62^. Amino acid sequences (Supplementary Data 1–3) were aligned with MAFFT^63^ and multiple sequence alignment were displayed and annotated with ESPript^64^. Secondary structure assignments were calculated with DSSP^65^. We used the PISA program to analyze subunit interfaces^66^. We obtained positional conservation scores from the multiple sequence alignments with the program ConSurf (v2016)^67^.

### Reporting summary

Further information on research design is available in the Nature Research Reporting Summary linked to this article.

## Supplementary information

Supplementary information

Description of Additional Supplementary Information File

Supplementary Movie 1

Supplementary Data 1

Supplementary Data 2

Supplementary Data 3

Reporting Summary

## Data Availability

The cryo-EM maps generated in this study have been deposited in the Electron Microscopy Data Bank under accession codes EMD-26603 (local reconstruction) and EMD-26602 (*N* = 38.5 helical reconstruction). The refined atomic coordinates generated in this study have been deposited in the Protein Data Bank under accession codes PDB-ID 7UML (local reconstruction) and PDB-ID 7UMK (*N* = 38.5 helical reconstruction). The previously published VSV structures used in this study are available in the Protein Data Bank under accession codes PDB-IDs 2GIC, 5UK4, and 1LG7). The VSV protein sequences used in this study to prepare the sequence alignments (shown in Supplementary Data 1–3) are available in the UniProt^68^ database under the following accession codes. The accession codes for the N protein sequences are: VSV_Indiana (P03523), vesicular stomatitis virus (Indiana strain); Morreton_virus (A0A0D3R1D1), Morreton vesiculovirus; Maraba_virus (F8SPF5), Maraba virus; Cocal_virus (B3FRL0), Cocal virus; Alagoas_virus (B3FRL5), vesicular stomatitis Alagoas virus; Carajas_virus (A0A0D3R1M8), Carajas virus; VSV_New_Jersey (P16379), vesicular stomatitis New Jersey virus; Chandipura_virus (P11211), Chandipura virus; Piry_virus (A0A1I9L1X2), Piry virus; Isfahan_virus (P16379), Isfahan virus; Rabies_virus (P06025), Rabies virus; Mokola_Virus (P0C570), Mokola virus. The accession codes for the M protein sequences are: VSV_Indiana (P03523), vesicular stomatitis virus (Indiana strain); Morreton_virus (A0A0D3R1D1), Morreton vesiculovirus; Maraba_virus (F8SPF5), Maraba virus; Cocal_virus (B3FRL0), Cocal virus; Alagoas_virus (B3FRL5), vesicular stomatitis Alagoas virus; Carajas_virus (A0A0D3R1M8), Carajas virus; VSV_New_Jersey (P16379), vesicular stomatitis New Jersey virus; Chandipura_virus (P11211), Chandipura virus; Piry_virus (A0A1I9L1X2), Piry virus; Isfahan_virus (P16379), Isfahan virus. All data are available from the corresponding authors upon reasonable request.

## Code availability

The software programs used to generate and analyze the data of this study are publicly available. Custom-written C shell and Python scripts used to run the programs and analyze data are available from the corresponding authors on reasonable request.
