# Supplementary material for: Visualizing molecular interactions that determine assembly of a bullet-shaped vesicular stomatitis virus particle
Source: Nat Commun. 2022 Aug 15;13:4802. doi: 10.1038/s41467-022-32223-1 (PMC9378655; doi:10.1038/s41467-022-32223-1)
Supplement: Supplementary file 1 — Supplementary information [file 41467_2022_32223_MOESM1_ESM.pdf]

SUPPLEMENTARY INFORMATION

Visualizing molecular interactions that determine assembly of a bullet-shaped vesicular stomatitis virus particle

Simon Jenni[1,*], Joshua A. Horwitz[1,2,5,6], Louis-Marie Bloyet[2,3,6], Sean P.J. Whelan[2,3], Stephen C. Harrison[1,4,*]

[1]Department of Biological Chemistry and Molecular Pharmacology, Harvard Medical School, Boston, MA 02115, USA

[2]Department of Microbiology, Harvard Medical School, Boston, MA 02115, USA

[3]Department of Molecular Microbiology, Washington University in St. Louis, St. Louis, MO 63110, USA

[4]Howard Hughes Medical Institute, Harvard Medical School, Boston, MA 02115, USA

[5]Present address: Molecular Pharmacology and Virology Group, PureTech Health LLC, Boston, MA 02210, USA

[6]These authors contributed equally

*Correspondence: jenni@crystal.harvard.edu, harrison@crystal.harvard.edu

Phone: 617-432-5601 Fax: 617-432-5600

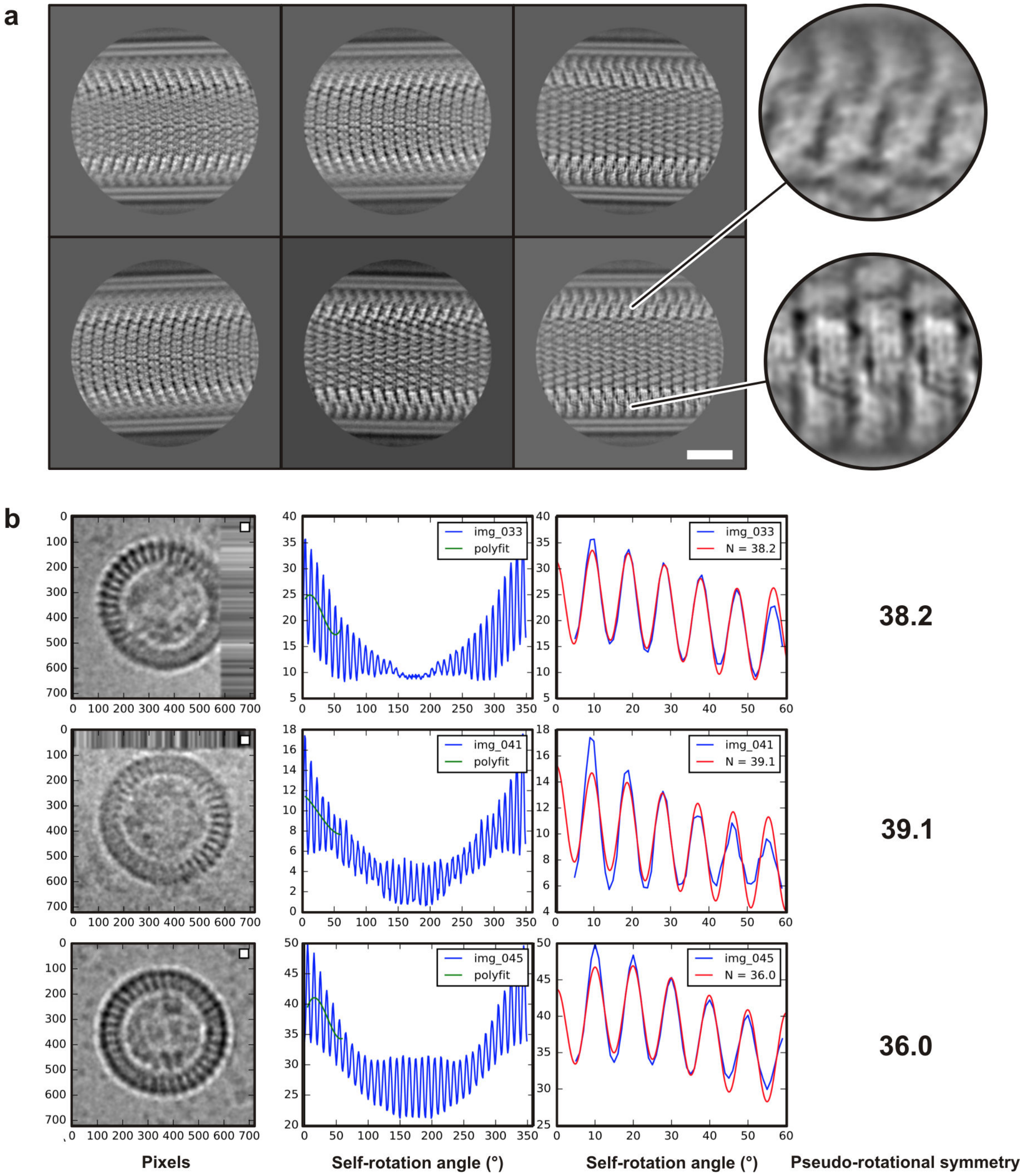

Supplementary Fig. 1 Heterogeneity of VSV helical nucleocapsid segments. a Representatives of best looking 2D class averages. Magnifications of two different regions of the bottom right class average show different degrees of sharpness, indicating structural heterogeneity and uneven focusing of the 2D alignment. The scale bar corresponds to 20 nm. **b** Three top-view images of partially assembled or disrupted virion fragments. We determined the apparent numbers of subunits per turn by fitting a cosine function (red curve) to the rotational self-correlation (blue curve) obtained from the observed density distribution within a radius of 153–255 Å. The green curve is a polynomial fit used for base line estimation.

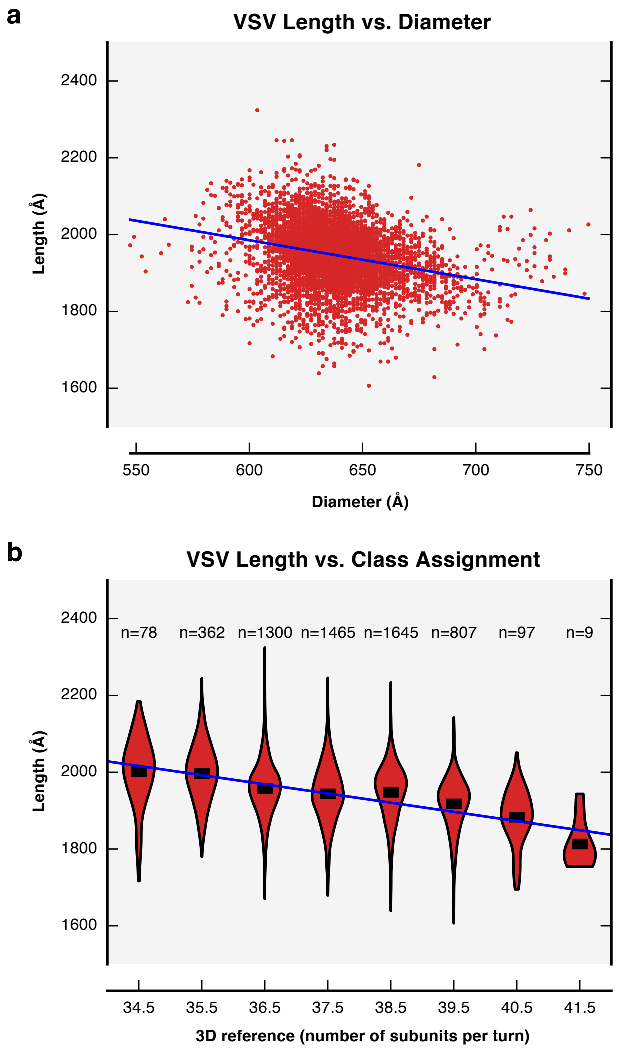

Supplementary Fig. 2 Structural heterogeneity of VSV virions. a Scatter plot of measured length versus diameter of individual VSV virions. The blue line is a linear regression fit to the data points (R-squared = 0.07, slope = -1.0). **b** Violin plot of measured length versus class assignment (after supervised classification) of individual virions. Length distributions and the number of virions and are shown for each class. Means are shown as horizontal black bars. The blue line is a linear regression fit to the means (R-squared = 0.89, slope = -23.9 per class).

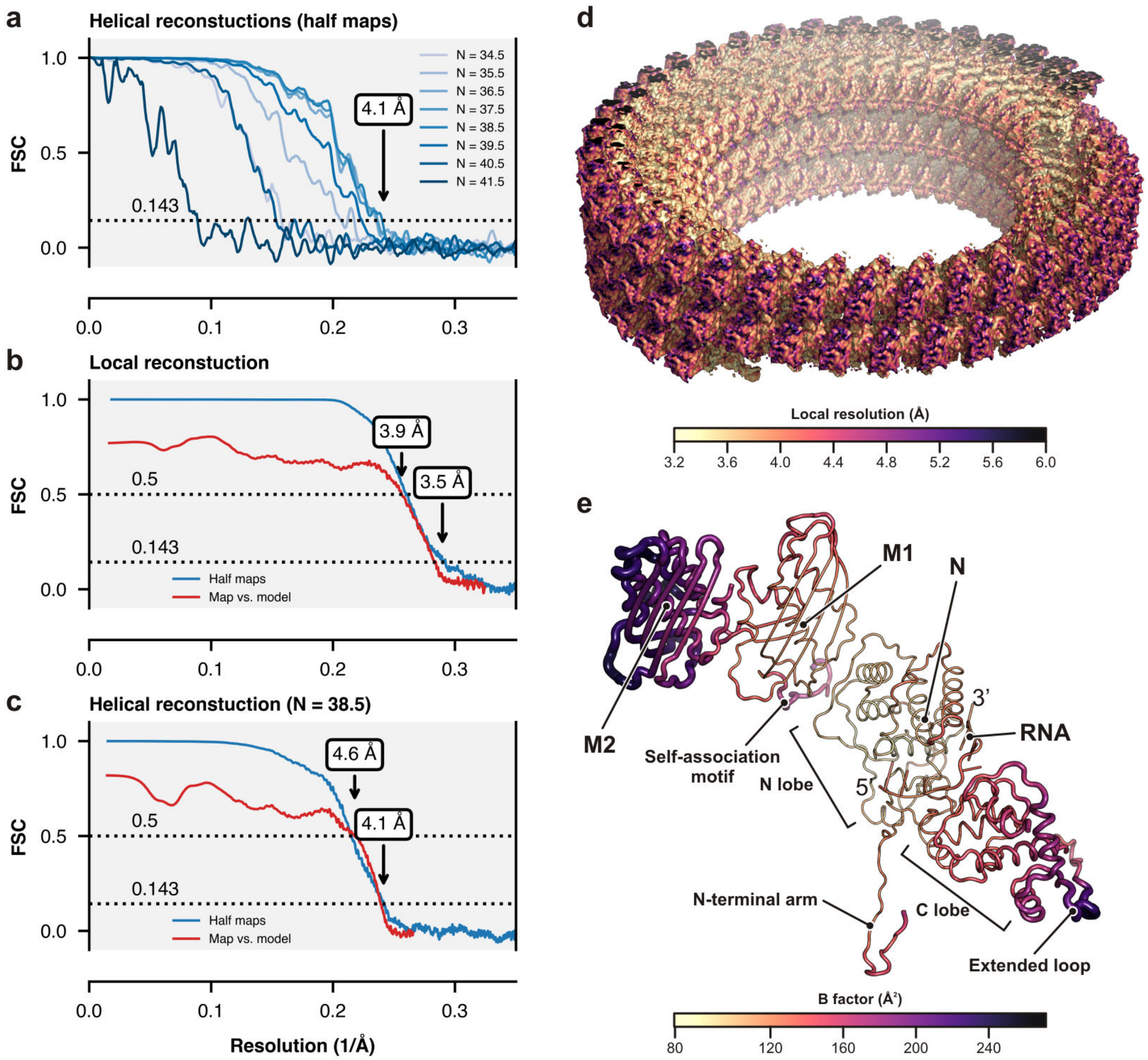

Supplementary Fig. 3 Resolution estimation of Cryo-EM reconstructions. a Top, Fourier shell correlation (FSC) between the two half maps for each of the helical reconstructions with different numbers of subunits per turn (*N*). Symmetrized half maps were masked with hollow cylindrical masks with inner and outer diameter as shown in Supplementary Table 1. The overall resolution where the correlation drops below 0.143 is 4.1 Å for the reconstruction with *N* = 38.5. **b** FSC analysis of the local reconstruction. The blue curve is the FSC between the half maps after masking based on the refined model. The red curve is the FSC between the final map and the refined model. The overall resolution is 3.5 Å for the half maps where the correlation drops below 0.143 and 3.8 Å for the model where the correlation drops below 0.5. **c** FSC analysis of the *N* = 38.5 helical reconstruction. The blue curve is the FSC between the half maps after masking based on the refined model. The red curve is the FSC between the final map and the refined model. The

overall resolution is 4.1 Å for the half maps where the correlation drops below 0.143 and 4.6 Å for the model where the correlation drops below 0.5. **d** Local resolution estimation color-mapped on the N = 38.5 helical reconstruction. **e** B factors mapped on the refined structure of the N = 38.5 helical reconstruction. Note that that B factor values are only meaningful relative within the structure as they depend on the degree of sharpening that was applied to the cryo-EM reconstruction.

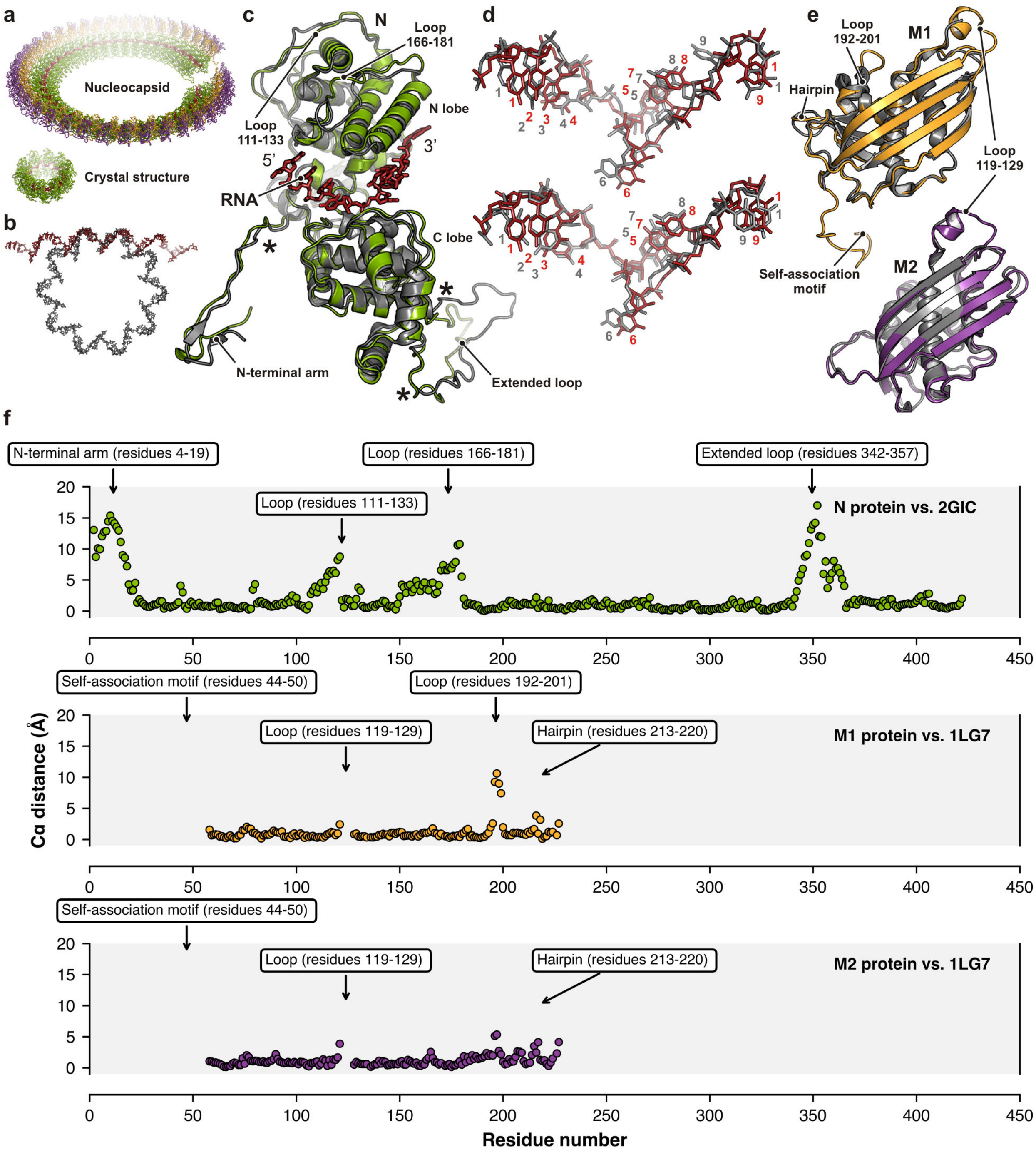

Supplementary Fig. 4 Structural comparison of nucleocapsid-assembled and crystallized RNA, N, and M proteins. a Overviews of the nucleocapsid (top, one turn of the N = 38.5 structure is shown) and crystallized decameric RNP complex (bottom) are shown. RNA is colored in red, N protein in green, M1 and M2 proteins in orange and purple, respectively. **b** Comparison of the RNA as observed in the nucleocapsid (red) and the crystal structure (gray). **c** Superposition of

the N protein from the nucleocapsid (green) and from the crystal structure (gray, PDB-ID 2GIC). Segments with substantially different conformations are labelled. Asterisks indicated flexibility points that allow the N-terminal arm and the extended loop to adjust for the different packing in the nucleocapsid and crystal structure. Residues of loop 111–133, solvent exposed or in lattice contacts in the crystal structures, interact with the M1 layer in the assembled nucleocapsid. Loop 166–181, mostly solvent exposed in the nucleocapsid, was incorrectly modeled in the crystal structures PDB-IDs 2GIC and 5UK4 (a sequence register shift). **d** Close-up view of the RNA structure from one repeating unit from the nucleocapsid (red) and the crystal structures (gray) PDB-ID 2GIC (top) and on PDB-ID 5UK4 (bottom) after superposition of the N proteins. Nucleotides are labeled 1–9. Note the different conformation of nucleotide 9. **e** Superposition of the M1 and M2 proteins from the nucleocapsid (orange and purple) and from the crystal structure (gray, PDB-ID 1LG7). Segments with substantially different conformations are labelled. The poorly ordered loop 119–129 in our density maps was not modeled in the crystal structure. M1 loop 192–201 and hairpin 213–220 bind the C terminus of the M2 subunits in the nucleocapsid. **f** The plots show the distances between corresponding Cα atoms after superposition of the N protein (green, calculated from residues 27–341 and 372–422) on PDB-ID 2GIC; the M1 protein (orange, calculated from residues 58–121 and 128-227) on PDB-ID 1LG7; the M2 protein (purple, calculated from residues 58–121 and 128-227) PDB-ID 1LG7. Regions with large conformational shifts are labeled on the top of each plot.

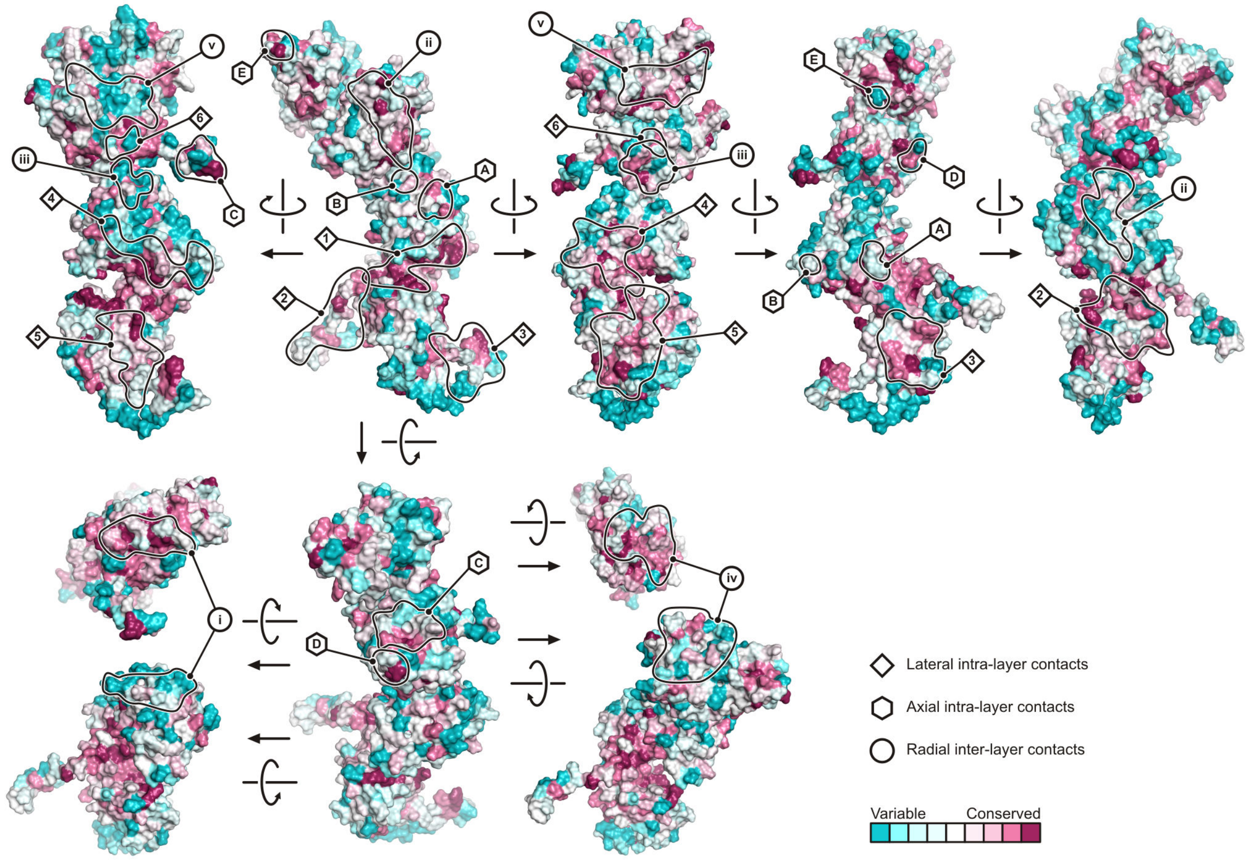

Supplementary Fig. 5 Conservation analysis of inter-subunit contacts. The N, M1, and M2 subunits of one module are shown in surface representation and colored according to amino acid conservation (see Supplementary Data 1–3 for multiple sequence alignments). Patches that form inter-subunit contacts are indicated and labeled as in Fig. 4.

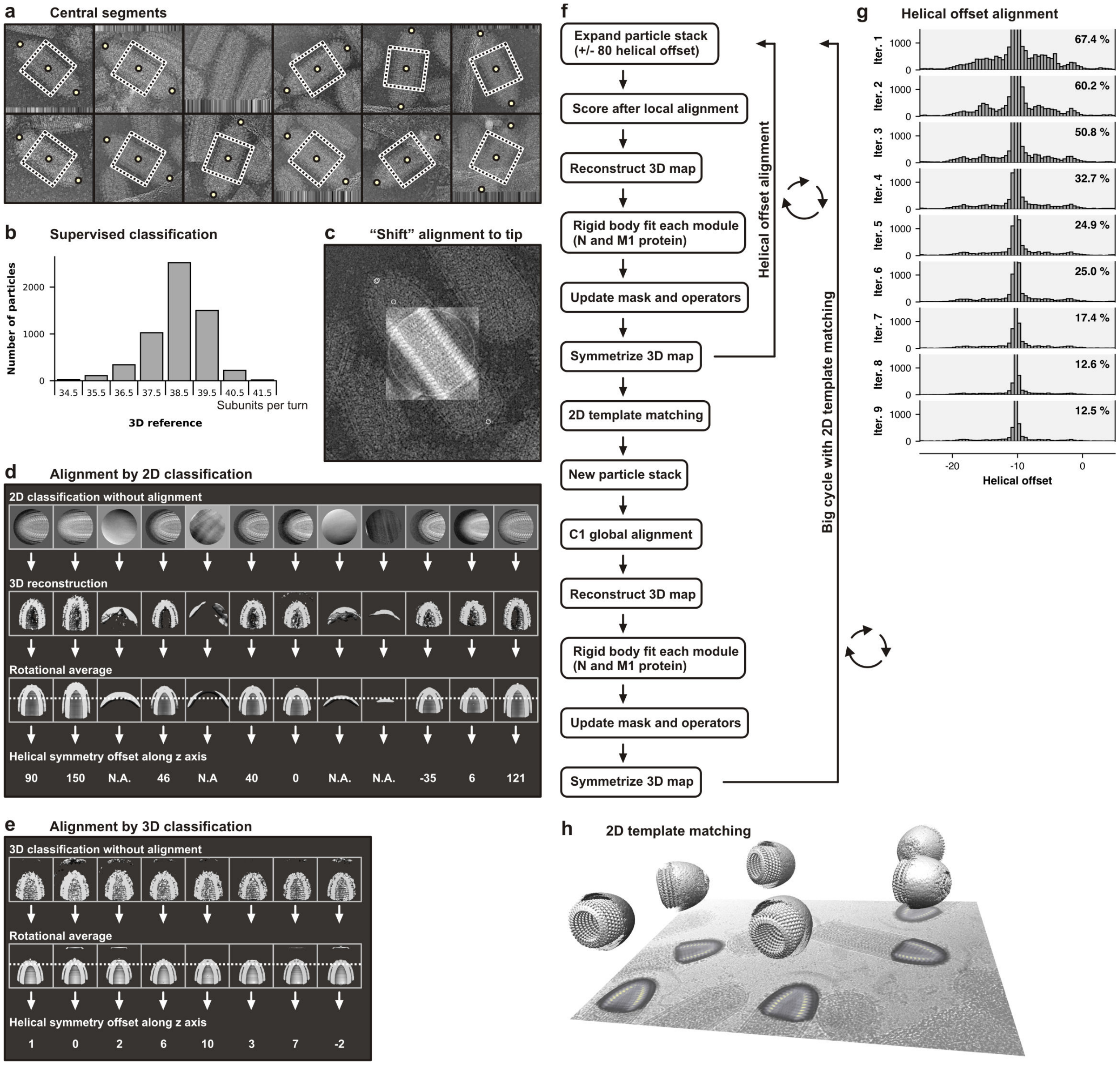

Supplementary Fig. 6 Reconstruction of the VSV tip. a Selection of central segment for initial alignment. The position for segment extraction was obtained after manually marking the top and bottom of virions in the micrographs (yellow dots). **b** Supervised classification of central segments. Only non-flattened 3D references with different numbers of subunits per turn were used for the analysis. **c** Shift of the alignment towards the VSV tip (see Supplementary Movie 1). **d** Alignment by 2D classification (without alignment). **e** Alignment by 3D classification (without alignment). **f** Flow chart showing the individual steps used to refine the reconstruction of the tip. The large cycle, which includes 2D template matching and global alignment, is computationally expensive. The small cycle (helical offset alignment) is much faster than the large cycle and was iterated to convergence. **g** Convergence of the small cycle (helical offset alignment). The histograms show

the distribution of particles with the best match to the current reference after applying a corresponding helical offset for each iteration. The percentage of particles with a helical offset is shown for each iteration. **h** 2D template matching illustrated for a representative micrograph.

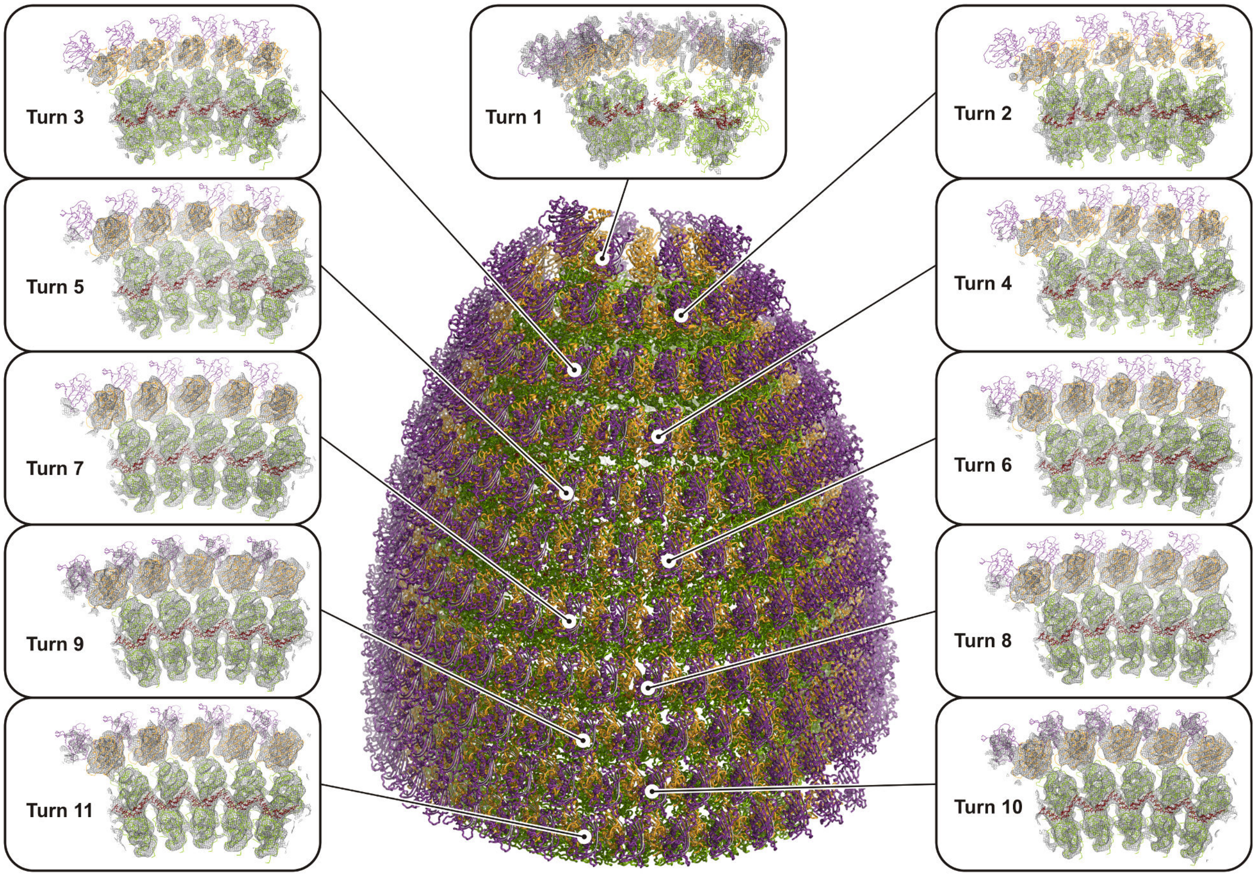

Supplementary Fig. 7 Structure of the VSV tip. In the center, a side view of the VSV tip structure is shown in ribbon representation. The nucleoprotein (N) is colored green, the RNA red, and the two matrix proteins (M1 and M2) are colored orange and purple, respectively. Modules comprised of one N, M1, and M2 proteins, and 9 nucleotides were fitted as rigid bodies. On the left and right, fits of the modules in the tip reconstruction density are shown for the first 11 turns of the RNP ribbon.

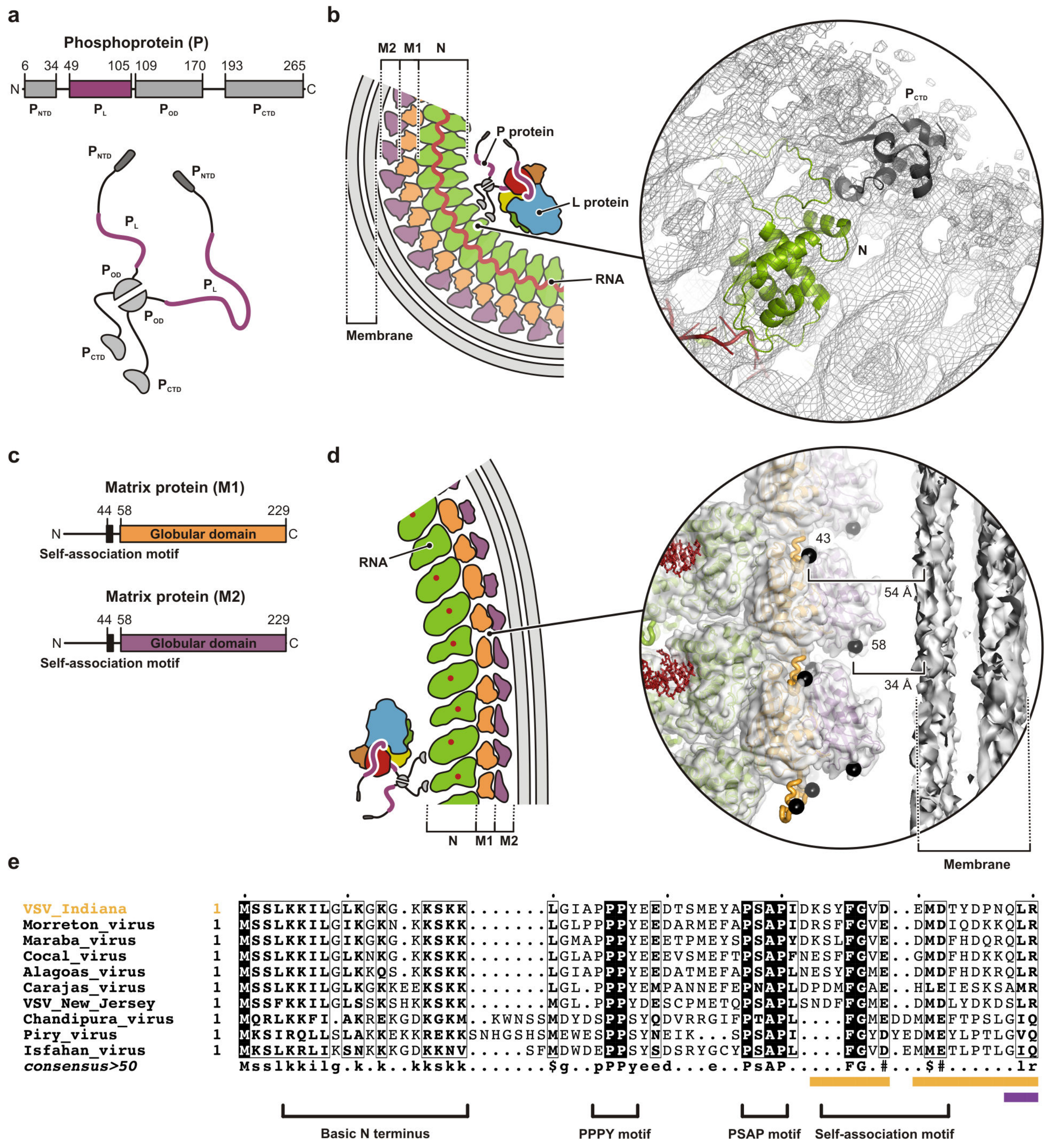

Supplementary Fig. 8 Binding of the phosphoprotein to the RNP and potential membrane-binding of the M protein. a Top, linear domain organization of the phosphoprotein (P). Amino acid numbers indicate domain boundaries. The P protein's N-terminal (P_{NTD}), oligomerization (P_{OD}), and C-terminal (P_{CTD}) domains are in gray; its L protein-binding domain (P_L) is in magenta. Bottom, schematic drawing of a P-protein dimer. **b** Density of the P protein's C-terminal (P_{CTD}, gray) domain bound to the N protein (green) was observed in low-pass filtered maps at low

contour levels. **c** Linear domain organization of the M1 and M2 proteins (as in Fig. 3a). **d** Distance between the first modelled residues of the M proteins (43 for M1, and 58 for M2) and the membrane of the VSV helical trunk. **e** Multiple sequence alignment of the M protein N terminus (residues 1–160 as in Supplementary Data 1). Modeled residues of the M1 and M2 are indicated at the bottom by orange and purple bars, respectively. Sequence motifs of interest are labeled.

Supplementary Table 1 Cryo-EM data collection and model statistics.

	Vesicular stomatitis virus (VSV, Indiana serotype)							
**Data collection**								
Electron microscope	Titan Krios							
Magnification	58823							
Voltage (kV)	300							
Defocus range (μm) [a]	1.0–3.0							
Pixel size (Å)	0.85							
Number of movies	18353							
**Helical reconstruction**	***N* = 34.5**	***N* = 35.5**	***N* = 36.5**	***N* = 37.5**	***N* = 38.5**	***N* = 39.5**	***N* = 40.5**	***N* = 41.5**
Number of images	1274	5393	17480	20367	21530	10369	1509	228
Box size (pixels)	1200	1200	1200	1200	1200	1200	1200	1200
Symmetry imposed	Helical	Helical	Helical	Helical	Helical	Helical	Helical	Helical
Helical twist (°)	-10.44	-10.14	-9.86	-9.60	-9.35	-9.11	-8.89	-8.67
Helical rise (Å)	1.488	1.449	1.411	1.374	1.341	1.309	1.276	1.248
Refinement mask diameter (Å) [b]	246, 546	258, 558	268, 568	280, 580	292, 592	302, 602	314, 614	326, 626
FSC mask diameter (Å) [b]	274, 534	286, 546	296, 556	308, 568	320, 580	330, 590	342, 602	354, 614
Map resolution (Å) [c]	6.9	4.9	4.3	4.2	4.1	4.5	6.6	12.1
**Local reconstruction**								
Number of images	2985175							
Box size (pixels)	256							
Symmetry imposed	C_1							
Map resolution (Å) [c]	3.5							
**Model statistics**	**Local reconstruction**				**Helical reconstruction *N* = 38.5**			
EMD accession identifier	EMD-26603				EMD-26602			
PDB accession identifier	7UML				7UMK			
Refinement resolution (Å)	3.8				4.1			
CC (mask)	0.70				0.75			
Model composition								
Non-hydrogen atoms	6750				6408			
Protein residues	812				778			
RNA nucleotides	13				9			
B factors (Å^2)								
Protein	44.6				157			
RNA	33.5				138			
R.m.s. deviations								
Bond lengths (Å)	0.003				0.002			
Bond angles (°)	0.575				0.534			
Validation								
MolProbity clash score	7.4				5.2			
Poor rotamers (%)	0.0				0.0			
Ramachandran plot								
Favored (%)	97.4				97.9			
Allowed (%)	2.0				1.6			
Disallowed (%)	0.6				0.5			

[a] Approximate range of underfocus.
[b] Inner and outer diameter of the cylindrical masks used for refinement and FSC calculation, respectively.
[c] Resolution where FSC between masked half maps drops below 0.143.
