# Supplementary material for: Visualizing molecular interactions that determine assembly of a bullet-shaped vesicular stomatitis virus particle
Source: Nat Commun. 2022 Aug 15;13:4802. doi: 10.1038/s41467-022-32223-1 (PMC9378655; doi:10.1038/s41467-022-32223-1)
Supplement: Supplementary file 2 — Description of Additional Supplementary Information File [file 41467_2022_32223_MOESM2_ESM.docx]

**Description of additional supplementary information files.**

**Supplementary Data 1 N Protein multiple-sequence alignment.**

N protein amino acid sequences from different viruses were obtained from UniProt with their sequence accession identifiers in parenthesis: VSV_Indiana (P03523), vesicular stomatitis virus (Indiana strain); Morreton_virus (A0A0D3R1D1), Morreton vesiculovirus; Maraba_virus (F8SPF5), Maraba virus; Cocal_virus (B3FRL0), Cocal virus; Alagoas_virus (B3FRL5), vesicular stomatitis Alagoas virus; Carajas_virus (A0A0D3R1M8), Carajas virus; VSV_New_Jersey (P16379), vesicular stomatitis New Jersey virus; Chandipura_virus (P11211), Chandipura virus; Piry_virus (A0A1I9L1X2), Piry virus; Isfahan_virus (P16379), Isfahan virus; Rabies_virus (P06025), Rabies virus; Mokola_Virus (P0C570), Mokola virus. Assigned secondary structure elements are annotated above the sequences. N protein residues that are within a distance of 5 Å to the RNA or neighboring N and M1 subunits in the structure of the *N* = 3.85 helical reconstruction are labeled below the sequences. Residues that contact the RNA are labeled with red ovals. Residues that contact N and M1 proteins are labeled with green and orange ovals, respectively.

**Supplementary Data 2 M1 Protein multiple-sequence alignment.**

M1 protein amino acid sequences from different viruses were obtained from UniProt with their sequence accession identifiers in parenthesis: VSV_Indiana (P03523), vesicular stomatitis virus (Indiana strain); Morreton_virus (A0A0D3R1D1), Morreton vesiculovirus; Maraba_virus (F8SPF5), Maraba virus; Cocal_virus (B3FRL0), Cocal virus; Alagoas_virus (B3FRL5), vesicular stomatitis Alagoas virus; Carajas_virus (A0A0D3R1M8), Carajas virus; VSV_New_Jersey (P16379), vesicular stomatitis New Jersey virus; Chandipura_virus (P11211), Chandipura virus; Piry_virus (A0A1I9L1X2), Piry virus; Isfahan_virus (P16379), Isfahan virus. M1 protein residues that are within a distance of 5 Å to neighboring N, M1, and M2 subunits in the structure of the *N* = 3.85 helical reconstruction are labeled below the sequences. Residues that contact N proteins are labeled with green ovals. Residues that contact M1 and M2 proteins are labeled with orange and purple ovals, respectively.

**Supplementary Data 3 M2 protein multiple-sequence alignment.**

M2 protein amino acid sequences from different viruses were obtained from UniProt with their sequence accession identifiers in parenthesis: VSV_Indiana (P03523), vesicular stomatitis virus (Indiana strain); Morreton_virus (A0A0D3R1D1), Morreton vesiculovirus; Maraba_virus (F8SPF5), Maraba virus; Cocal_virus (B3FRL0), Cocal virus; Alagoas_virus (B3FRL5), vesicular stomatitis Alagoas virus; Carajas_virus (A0A0D3R1M8), Carajas virus; VSV_New_Jersey (P16379), vesicular stomatitis New Jersey virus; Chandipura_virus (P11211), Chandipura virus; Piry_virus (A0A1I9L1X2), Piry virus; Isfahan_virus (P16379), Isfahan virus. M2 protein residues that are within a distance of 5 Å to neighboring M1 and M2 subunits in the structure of the *N* = 3.85 helical reconstruction are labeled below the sequences. Residues that contact M1 and M2 proteins are labeled with orange and purple ovals, respectively.

**Supplementary Movie 1 VSV tip reconstruction.**

Shift of the alignment towards the VSV tip. A projection of the 3D reference according to the alignment parameters is mapped onto the virion image. The two small circles at the virion tip are (i) the tip initially marked manually in the micrographs and (ii) its projection onto the projection of the helical axis of the aligned 3D reference. The small circle with a dot is the projected center of the reference box. The small circle in between is a projected defined point on the z axis (helical axis) of the reference box. The movie shows how the alignment shifts from an initial central segment towards the tip of the virus. The reference was shifted according to helical symmetry and the alignment parameters were updated at each position by local refinement until the distance between the small circle (ii) and the projection of defined point on the z axis was a minimum.
