# Supplementary material for: Visualizing molecular interactions that determine assembly of a bullet-shaped vesicular stomatitis virus particle
Source: Nat Commun. 2022 Aug 15;13:4802. doi: 10.1038/s41467-022-32223-1 (PMC9378655; doi:10.1038/s41467-022-32223-1)
Supplement: Supplementary file 4 — Supplementary Data 1 [file 41467_2022_32223_MOESM4_ESM.pdf]

```
N_protein

VSV_Indiana       1  MSVTVKRIIDNTVIVPKLP...ANEDPVEYPADYF.RKSKEIPLYINTTKSLSDLRGYVYQGLKSGNVSI
Morreton_virus    1  MSVTVKRIIDNSVILPKLP...ANEDPVEYPGDYF.KKTNEVPVYINTSKTLNDLRGYVYQGLKTGNVSI
Maraba_virus      1  MSVTVKRVIDDSLITPKLP...ANEDPVEYPADYF.KKSRDIPVYINTTKSLSDLRGYVYQGLKSGNISI
Cocal_virus       1  MAATVKRVIDDSVITPKLP...ANEDPVEYPADYF.KKSRDIPVYINTTKSLADLRGYVYQGLKAGSLSI
Alagoas_virus     1  MAAVVKRIIDDTLIVPKLP...ASEDPVEYPADYF.KKTSEIPLFINTTKSLAELRGFVYQGLKSGVVSI
Carajas_virus     1  MNSIVKKVIDDTVIQPKLP...ANEDPVEYPADYF.KTSKQIPLYINTDKTLAELRAFVYQGLKAGNPSI
VSV_New_Jersey    1  MAPTVKRIINDSIIQPKLP...ANEDPVEYPADYF.KNNTNIVLYVSTKVALNDLRAYVYQGIKSGNPSI
Chandipura_virus  1  MSSQVFCISTGQTVSVCLP...ANEDPVEFPGAFFTPNAKKPTVYIKKETDLSLLRSHVYDGIKDGSVTV
Piry_virus        1  MSVSVKRISTGTQVVAALP...ASEDPVEFPAEYFAKNPGKIPVFISTRLDLDKLRQYVYMGLTTGEVNV
Isfahan_virus     1  MTSVVKRIATGSSVLAVLP...ANEDPVEFPGDYFLQNPGKIRVCINRKLDVATLRQYVYEGLKNGDVHV
Rabies_virus      1  MDADKIVFKVNNQVVSLKPEIIVDQYEYKYPAI...KDLKKPCITLGKAPDLNKAYKSVLSCMSAAKLDP
Mokola_virus      1  MESDKIVFKVNNQVVSLKPEVISDQYEYKYPAI...LDGKKPGITLGKAPDLNTAYKSILSGMKAAKLDP
consensus>50         M...vk.i..d..!...lP...an#dpve%Padyf......i..yi.....l..lr.y!yqglk.g...i

N_protein

VSV_Indiana      67  IHVNSYLYGALKDIRGKLDKDWSSFGINIGKAGDTIGIFDLVSLK..ALDGV.LPDGVSDASR..TSADD
Morreton_virus   67  IHVNSYLYAALRDIKGKLDKDWISFGVQIGKTGDEVGIFNLVSVK..TLEGI.IPDGVSDASR..TSADD
Maraba_virus     67  IHVNSYLYAALKEIRGKLDRDWITFGIQIGKTGDSVGIFDLLTLK..PLDGV.LPDGVSDATR..TSSDD
Cocal_virus      67  VHVNSYLYAALKDVKGKLDRDWITFNIQIGKAGDNVGIFDLVTVK..NLEGV.CPDGVSDATR..TSADD
Alagoas_virus    67  IHVNSYLYAALKDVQAKLDRDWTSFGVNIGRAGANVGIFDLVSVK..GLEGV.CPDGVSDASR..TSADD
Carajas_virus    67  IHVNSYLYLALKDIKATLERDWTSFSITIGKQGEEITIFNLVSVR..PLVIT.VPDGRTDPDR..SPNDD
VSV_New_Jersey   67  LHINAYLYAALKGVEGTLDRDWVSFGRTIGKREENVKIFDLVKVE..ELKTA.LPDGKSDPDR..SAEDD
Chandipura_virus 68  SQINSYLYMVLKDIREKPDKNWTSFGVELGKKNEPMGIFDLLNVE..DVKGK.ELDKKGQDTR..LPGDD
Piry_virus       68  CHINSYLYRVLQNIEDEARDNWVSFRVTIAAKDTTVRLFDMLEVK..EYAGL.VPDGKENANL..TQTVD
Isfahan_virus    68  CHINSYLYQVLKDTRDEAQSDWISFGVSLAVKGGIVSVFDTLMIE..DYRGE.APDGRKCDGR..TIDDD
Rabies_virus     68  DDVCSYLAAAMQFFEGTCPEDWTSYGIVIARKGDKITPGSLVEIKRTDVEGNWALTGGMELTRDPTVPEH
Mokola_virus     68  DDVCSYLAAAMHLFEGVCPEDWVSYGIVIAKKGEKINPSVIVDIVRTNVEGNWAQAGGTDVIRDPTMAEH
consensus>50         .h!nsYLy.a$.di....d.#W.s%gi.i...gd.v.ifdl..v......g...pdg..d..r..t..dd

N_protein

VSV_Indiana     132  KWLPLYLLGLYRVGR...TQMPEYRKKLMDGLTNQCKMINEQFEPLVPEGRDIFDV..............
Morreton_virus  132  AWLPLYLLGLYRVGR...TQMPEYRKKLMDGLINQCKMINEKFEPLVPEGRDIFDV..............
Maraba_virus    132  AWLPLYLLGLYRVGR...TQMPEYRKKLMDGLINQCKMINEQFEPLLPEGRDVFDV..............
Cocal_virus     132  QWLPLYLLGLYRVGR...TQMTDYRKKLMDGLIAQCKLINEKFEPLIPEGRDFYDI..............
Alagoas_virus   132  NWLPLYLLGLYRVGR...TQMTDYRKRLMDGLINQCKMIDDKFEPLIPEGRDFFDL..............
Carajas_virus   132  KWLPIYLLGLYRVGR...TKLPEYRKKLMEGLEMQCKIMYPDFVPIVPEGMDFFDV..............
VSV_New_Jersey  132  KWLPIYILGLYRVGR...SKVTDYRKKLLDGLENQCRVASTRFESLVEDGLDFFDI..............
Chandipura_virus 133 LWLPTLIFGLYRVSR...ATQVEYKKTLMTNLYAQCKLRTKDAEEIVDETAEFFNA..............
Piry_virus      133  EWLPMYILGLQRVGR...ATDERYRGDLYDHLCAQCKLKSPSFESLGNPADNNYAS..............
Isfahan_virus   133  KWLPMLILGLYRVSR...ATQEDYKKSLLQKLYAQCKLRSPQAEELVEDAAEFYEV..............
Rabies_virus    138  ASLVGLLLSLYRLSKISGQSTGNYKTNIADRI............EQIFETAPFVKIVEHHTLMTTHKMCA
Mokola_virus    138  ASLVGLLLCLYRLSKIVGQNTANYKTNVADRM............EQIFETAPFAKIVEHHTLMTTHKMCA
consensus>50         .wLp...lgLyRv.r.......eY.k.lmd.l..qck......e..v.e..d.fdv..............

N_protein

VSV_Indiana     185  .WGNDSNYTKIVAAVDMFFHMFKKHECASFRYGTIVSRFKDCAALATFGHLCKITGMSTEDVTTWILNRE
Morreton_virus  185  .WGNDSNYTKIVAAVDMFFHMFKKHEKASFRYGTIVSRFKDCAALATFGHLCKITGMSTEDVTTWILNRE
Maraba_virus    185  .WGNDSNYTKIVAAVDMFFHMFKKHEKASFRYGTIVSRFKDCAALATFGHLCKITGMSTEDVTTWILNRE
Cocal_virus     185  .WGNDGNYTKIVAAVDMFFHMFKKHEKASFRYGTIVSRFKDCAALATFGHVCKITGMSTEEVTTWILNRE
Alagoas_virus   185  .WGNDNNYTKIIAAVDMFFHMFKKHERAYLRYGTIVSRFKDCAALATFGHVCKITGMSTEEVTTWILNRE
Carajas_virus   185  .WGNDSNFTKIVAAVDMFFHMFKKHERASLRYGTIVSRFKDCAALATFGHVCKVSGMSTEEVTTWVLNRE
VSV_New_Jersey  185  .WENDPNFTKIVAAVDMFFHMFKKHERAPIRYGTIVSRFKDCAALATFGHLSKVSGLSIEDLTTWVLNRE
Chandipura_virus 186 .WANDSNFTKIVAAVDMYFHHFKKSDHAPIRFGTIVSRFKDCAALSTLSHLQKVTGLPIEEVFTWVFNKS
Piry_virus      186  .WINDSNFLKLVAAIDMFFHHFKKHEEAVIRFGTITSRFKDCAALSTMAHLVKVTGMPVEEVMTWVLNKP
Isfahan_virus   186  .WSNDSNFLKLVAAIDMFFHKFKNHADAGLRWGTIVSRFKDCAALATLSHVQKVTGLSIKEVFTWVLNKS
Rabies_virus    196  NWSTIPNFRFLAGTYDMFFSRI.EHLYSAIRVGTVVTAYEDCSGLVSFTGFIKQINLTAREAILYFFHKN
Mokola_virus    196  NWSTIPNFRFLVGTYDMFFARV.EHIYSALRVGTVVTAYEDCSGLVSFTGFIKQINLSPRDALLYFFHKN
consensus>50         .W.nd.N%.k.vaavDM%Fh.fkkhe.a..RyGT!vsr%kDCaaL.tf.h..Ki.g$s.e#v.twiln.e
```

N_protein

```
VSV_Indiana      254  VADEMVQMMLPGQEIDKADSYMPYLIDFGLSSKSPYSSVKNPAFHFWG..QLTALLLRSTRARNARQPDD
Morreton_virus   254  VADEMVQMMLPGQEIDKADSYMPYLIDFGLSSKSPYSSVKNPAFHFWG..QLTALLLRSTRARNARQPDD
Maraba_virus     254  VADEMVQMMYPGQEIDKADSYMPYLIDLGLSSKSPYSSVKNPAFHFWG..QLTALLLRSTRARNARQPDD
Cocal_virus      254  VADEMVQMMYPGQEIDKSDSYMPYLIDLGLSQKSPYSTVKNPAFHFWG..QLTALLLRSTRARNARQPDD
Alagoas_virus    254  VGDEMIQMMKPGQEIDKADSYMPYLIDFGLSQKSPYSTVKNPAFHFWG..QLTALLLRSTRAKNARQPDD
Carajas_virus    254  VADELCQMMFPGQEIDRADSYMPYMIDFGLSQKSPYSSVKNPSFHFWG..QLAALLLRSTRAKNARQPDD
VSV_New_Jersey   254  VADELCQMMYPGQEIDKADSYMPYMIDFGLSQKSPYSSVKNPAFHFWG..QLAALLLRSTRAKNARQPDD
Chandipura_virus 255  VQDDLLRMMTPGQEIDQADSYMPYLIDMGLSTKSPYSSTKNPSFHFWG..QLTAFLVKSARAKNALVPVD
Piry_virus       255  VEDEVCRMMTPGQEIDKADSYMPYLIDFGLSTKSPYSSVKNPCFHFWG..QLTAVLVNSARARNARVPDD
Isfahan_virus    255  VEDELCRMMKERQEVDKADSYMPYLIDFGISTKSPYSSVKNPCFHFWG..QLTALLVHSHRAKNARVPED
Rabies_virus     265  FEEEIRRMFEPGQETAVPHSYFIHFRSLGLSGKSPYSS..NAVGHVFNLIHFVGCYMGQVRSLNATVIAA
Mokola_virus     265  FEGEIKRMFEPGQETAVPHSYFIHFRALGLSGKSPYSS..NAVGHTFNLIHFVGCYMGQIRSLNATVIQT
consensus>50          v.d#m..Mm.pgQEid.adSYmpylid.GlS.KSPYSsvkNp.fHfwg..ql.a.ll.s.Ra.NAr.pdd
```

N_protein

```
VSV_Indiana      322  IEYTSLTTAGLLYAYAVGSSADLAQQFCVGDNKYTPD.....DSTGGLTTNAPPQGRDVVEWLGWFEDQN
Morreton_virus   322  IEYTSLTTAGLLYAYAVGSSADLAQQFCIGDNKYVPD.....PNAGGLTTNAPPQGRDVVEWLGWFEDQN
Maraba_virus     322  IEYTSLTTAGLLYAYAVGSSADLAQQFYVGDNKYVPE.....TGDGGLTTNAPPQGRDVVEWLSWFEDQN
Cocal_virus      322  IEYTSLTTAGLLYAYAVGSSADLSQQFYVGDDKYISE.....NTTGGLNSNAPPQGRDVVEWLGWFDDNQ
Alagoas_virus    322  IEYTSLTTAGLLYAYAVGSSADLTQQFYMGDNKYVPD.....NSDGGLTTNAPPQGRDVVEWLGWFDDNH
Carajas_virus    322  IEYTSLTTAGLLLAYAVGSSADISQQFYMGDEKYISD.....PSAGGLTSNAPPKGRNVVDWLGWFEDQG
VSV_New_Jersey   322  IEYTSLTCASLLSFAVGSSADIEQQFYIGEDKYTTE.....KDDSLKKSDVPPKGRNVVDWLGWYDDNG
Chandipura_virus 323  IAYHELTTAALLFAYAIGRSSELEQRFVLNGKKFTKE.....KDSREDNDTTPPSERNVVVWLAWWEDIK
Piry_virus       323  IPYQELTQAALLFAYATGRSSDLAQRFVLHNKEYIKAAPDASDNSEREESKEAPTTRDVVEWLAWWDDIG
Isfahan_virus    323  IPYNELTTAAWLFAYAMGRSSGLEQRFTTDDSYYQED.....EDINKGLGVKAPTTRDVQMWLAWWSDIG
Rabies_virus     333  CAPHEMSVLGGYLGEEFFGKGTFERRFFRDEKELQEY.....EAAELTKTDVALADDGTVN..SDDEDYF
Mokola_virus     333  CAPHEMSVLGGYLGEEFFGKGTFERRFFRDEKEMQDY.....TELEEARVEASLADDGTVD..SDEEDFF
consensus>50          i.y..$t.a.ll.aya.g.s.d..q.F...d..y..d............n..p..rdvvewl.w.eD..
```

N_protein

```
VSV_Indiana      387  RKPTPDMMQYAKR.................AVMSLQGLREKTIGKYAKSEFDK..
Morreton_virus   387  RKPTPDMLLYAKR.................AVNSLQGLREKTVGKYAKSEFDK..
Maraba_virus     387  RKPTPDMLMYAKR.................AVSALQGLREKTIGKYAKSEFDK..
Cocal_virus      387  RKPTADMLQYAKK.................AVMSLQGLRDKTIGKYAKAEFDK..
Alagoas_virus    387  RKPTPDMLQYAKR.................AVMSLQGLRDKTIGKYAKSEFDK..
Carajas_virus    387  GNITPDMYSFAKR.................AVCSLQGLRDKTIGKYAKGEFDK..
VSV_New_Jersey   387  GKPTPDMLNFARR.................AVSSLQSLREKTIGKYAKVEFDK..
Chandipura_virus 388  HEITPDMKAFAKR.................AVERVGDIRVNSVAEYARKLFA...
Piry_virus       393  QVRTRDMEVFARR.................AVHGILDPREKSIGAYARGYFA...
Isfahan_virus    388  KVPTQDMETFARR.................EVLGLTEIRSKTIGEYAKKTFSV..
Rabies_virus     396  SGETRSPEAVYTRIIMNGGRLKRSHIRRYVSVSSNHQARPNSFAEFLNKTYSSDS
Mokola_virus     396  SGETRSPEAVYSRIMMNNGKLKKVHIRRYIAVSSNHQARPNSFAEFLNKVYADGS
consensus>50          ...T.dm..ya.r.................aV......R.k.ig.%a...%....
```
