# Supplementary material for: Visualizing molecular interactions that determine assembly of a bullet-shaped vesicular stomatitis virus particle
Source: Nat Commun. 2022 Aug 15;13:4802. doi: 10.1038/s41467-022-32223-1 (PMC9378655; doi:10.1038/s41467-022-32223-1)
Supplement: Supplementary file 5 — Supplementary Data 2 [file 41467_2022_32223_MOESM5_ESM.pdf]

```
M1_protein

VSV_Indiana         1   MSSLKKILGLKGKG.KKSKK.......LGIAPPPYEEDTSMEYAPSAPIDKSYFGVD..EMDTYDPNQLR
Morreton_virus      1   MSSLKKILGIKGKN.KKSKK.......LGLPPPPYEEDARMEFAPSAPIDRSFFGVE..DMDIQDKKQLR
Maraba_virus        1   MSSLKKILGIKGKG.KKSKK.......LGMAPPPYEEETPMEYSPSAPYDKSLFGVE..DMDFHDQRQLR
Cocal_virus         1   MSSLKKILGLKNKG.KKSKK.......LGLAPPPYEEEVSMEFTPSAPFNESFFGVE..GMDFHDKKQLR
Alagoas_virus       1   MSSLKKILGLKKQS.KKSKK.......LGIAPPPYEEDATMEFAPSAPLNESYFGME..DMDFHDKRQLR
Carajas_virus       1   MSSLKKILGLKGKKEEKSKK.......LGL.PPPYEMPANNEFEPNAPLDPDMFGAE..HLEIESKSAMR
VSV_New_Jersey      1   MSSFKKILGLSSKSHKKSKK.......MGL.PPPYDESCPMETQPSAPLSNDFFGME..DMDLYDKDSLR
Chandipura_virus    1   MQRLKKFI.AKREKGDKGKM..KWNSSMDYDSPPSYQDVRRGIFPTAPL....FGMEDDMMEFTPSLGIQ
Piry_virus          1   MKSIRQLLSLAKKEKKREKKSNHGSHSMEWESPPSYNEIK...SPSAPI....FGYDYEDMEYLPTLGVQ
Isfahan_virus       1   MKSLKRLIKSNKKKGDKKNV.....SFMDWDEPPSYSDSRYGCYPSAPL....FGVD.EMMETLPTLGIQ
consensus>50            Msslkkilg.k.k..kkskk.......$g..pPPyeed...e..PsAP.....FG.#...$#......lr
```

```
M1_protein

VSV_Indiana        61   YEKFFFTVKMTVRSNRPFRTYSDVAAAVSHWDHMYIGMAGKRPFYKILAFLGSSNLKATPAVLADQGQPE
Morreton_virus     61   YEKFYFSVKMTVRSNRPFRTYSDVASAVSNWDHMYIGMAGKRPFYKILAFLGSTLLKATPAVLADHGQPE
Maraba_virus       61   YEKFHFSLKMTVRSNKPFRNYDDVAAAVSNWDHMYIGMAGKRPFYKILAFMGSTLLKATPAVLADQGQPE
Cocal_virus        61   HEKFHFSVKLTVQSNRPFRTYDDVVAAVSNWDHMYIGMAGKRPFYKVLAFLASTHLRATPAVLADQGHPE
Alagoas_virus      61   YEKFHFSIKLTVQSNRPFRSCEDAMIAVSNWDHMYIGLAGKRPFYKVLALLGSTLLKATPAVLADQGHPE
Carajas_virus      61   YEKFKFSVKITLRTNRPLRTYDDVCQILSKWDAMYVGMMGKRPFYKVLVLIGSSHLQATPAILSDRGQPE
VSV_New_Jersey     61   YEKFRFMLKMTVRSNKPFRSYDDVTAAVSQWDNSYIGMVGKRPFYKIIAVIGSSHLQATPAVLADLNQPE
Chandipura_virus   64   TLKLQYKCVVNINAINPFRDFREAISAMQFWEADYSGYIGKKPFYRAIILHTARQLKTSNPGILDRGVVE
Piry_virus         64   TLKLQYKCVLQVRSESPFTSYLDAVDNVANWEKQYNGFSGKKPFYRAVMVRAVQAMKANPMSLQDGRSPE
Isfahan_virus      61   SLKIQYKCSLQVRAETPFTSFNDAARCISLWETDYRGYAGKKPFYRLLMLIATKKLRAAPMSLMDGNRPE
consensus>50            .eKf.%..kmtvrsn.Pfr.y.#...avs.W#..YiGm.GKrPFYkil....s..$.atpavl.D.g.pE
```

```
M1_protein

VSV_Indiana       131   YHTHCEGRAYLPHRMGKTPPMLNVPEHFRRPFNIGLYKGTIELTMTIYDDESLEAAPMIWDHFNSSKFSD
Morreton_virus    131   YHAHCEGRAYLPHRLGPTPPMLNVPEHFRRPFNIGLFRGTIDLTMTLHDDESLEAAPMIWDHFNASRVTD
Maraba_virus      131   YHAHCEGRAYLPHRLGPTPPMLNVPEHFRRPFNIGLFRGTIDITLVLFDDESVDSAPVIWDHFNASRLSS
Cocal_virus       131   YHAFCEGRAYLPHRLGATPPMLNVPEHFRRPFHIGLYRGTVDLTVTLFDDESVDSANMIWDHFNSSRSYN
Alagoas_virus     131   YHAFCEGRAYLPHRLGPTPPMLNVPEHFRRPFHIGLYRGTVDLTLTLYDDESTESAPVIWDYINSSRVKN
Carajas_virus     131   YHMYLEDRGFIAHRLGLTPPMLSGPESFRRPFHVGLYRGTIDITVNLMDDESTESAPQVWDHFNTRYVNH
VSV_New_Jersey    131   YYATLTGRCFLPHRLGLIPPMFNVQETFRKPFNIGLYKGTLDFTFTVSDDESNEKVPHVWDYMNPKYQSQ
Chandipura_virus  134   YHATTQGRALVFHSLGPSPSMMFVPETFTREWNILTNKGTINVKIWLGETDTLSELEPILNPVNFRDDRE
Piry_virus        134   YTSEIEGRCLVFHSLGHIPPMMYMCEQFTRDWSGRRNQGIVNVKIWVGVTDTLDNLDQIFDPKKHFSEEE
Isfahan_virus     131   YSSMIQGQSIVHHSLGVIPPMMHVPETFTREWNLLTNKGMITAQIWLGITDVVDDLNPLINPALFSDEKE
consensus>50            Yh...egr.y..Hr$G..PpMl.vpE.Frrpf.igly.Gtid.t..l.dd#s.e....iw#..n.....e
```

```
M1_protein

VSV_Indiana       201   FREKALMFGLIVEKKASGAWVLDSISHFK
Morreton_virus    201   FPEKALLFGLIVEKKATGAWILDSISNFK
Maraba_virus      201   FREKALLFGLILEKKATGNWVLDSISHFK
Cocal_virus       201   MKEKALLFGLILERKATGSWVLDSISNFK
Alagoas_virus     201   LRENALMFGLIVERKATGSWVLDSISHFK
Carajas_virus     201   FLEHAKRFGLVLSKKPGGGWILDQ.AVCA
VSV_New_Jersey    201   IQQEGLKFGLILSKKATGTWVLDQLSPFK
Chandipura_virus  204   MIEGAAIMGLEIKKQKDNTWLISK.SH..
Piry_virus        204   MLSAATILGLEVKKSSDNNYIISK.SY..
Isfahan_virus     201   MTLTSQMFGLELKKRNDNTWLISK.SY..
consensus>50            ..e.a..fGLi..kk..g.wvld..s...
```
