# Supplementary material for: Visualizing molecular interactions that determine assembly of a bullet-shaped vesicular stomatitis virus particle
Source: Nat Commun. 2022 Aug 15;13:4802. doi: 10.1038/s41467-022-32223-1 (PMC9378655; doi:10.1038/s41467-022-32223-1)
Supplement: Supplementary file 6 — Supplementary Data 3 [file 41467_2022_32223_MOESM6_ESM.pdf]

```
M2_protein

VSV_Indiana        1  MSSLKKILGLKGKG.KKSKK.......LGIAPPPYEEDTSMEYAPSAPIDKSYFGVD..EMDTYDPNQLR
Morreton_virus     1  MSSLKKILGIKGKN.KKSKK.......LGLPPPPYEEDARMEFAPSAPIDRSFFGVE..DMDIQDKKQLR
Maraba_virus       1  MSSLKKILGIKGKG.KKSKK.......LGMAPPPYEEETPMEYSPSAPYDKSLFGVE..DMDFHDQRQLR
Cocal_virus        1  MSSLKKILGLKNKG.KKSKK.......LGLAPPPYEEEVSMEFTPSAPFNESFFGVE..GMDFHDKKQLR
Alagoas_virus      1  MSSLKKILGLKKQS.KKSKK.......LGIAPPPYEEDATMEFAPSAPLNESYFGME..DMDFHDKRQLR
Carajas_virus      1  MSSLKKILGLKGKKEEKSKK.......LGL.PPPYEMPANNEFEPNAPLDPDMFGAE..HLEIESKSAMR
VSV_New_Jersey     1  MSSFKKILGLSSKSHKKSKK.......MGL.PPPYDESCPMETQPSAPLSNDFFGME..DMDLYDKDSLR
Chandipura_virus   1  MQRLKKFI.AKREKGDKGKM..KWNSSMDYDSPPSYQDVRRGIFPTAPL....FGMEDDMEFTPSLGIQ
Piry_virus         1  MKSIRQLLSLAKKEKKREKKSNHGSHSMEWESPPSYNEIK...SPSAPI....FGYDYEDMEYLPTLGVQ
Isfahan_virus      1  MKSLKRLIKSNKKKGDKKNV.....SFMDWDEPPSYSDSRYGCYPSAPL....FGVD.EMMETLPTLGIQ
consensus>50          Msslkkilg.k.k..kkskk.......$g..pPPyeed...e..PsAP.....FG.#...$#......lr

M2_protein

VSV_Indiana       61  YEKFFFTVKMTVRSNRPFRTYSDVAAAVSHWDHMYIGMAGKRPFYKILAFLGSSNLKATPAVLADQGQPE
Morreton_virus    61  YEKFYFSVKMTVRSNRPFRTYSDVASAVSNWDHMYIGMAGKRPFYKILAFLGSTLLKATPAVLADHGQPE
Maraba_virus      61  YEKFHFSLKMTVRSNKPFRNYDDVAAAVSNWDHMYIGMAGKRPFYKILAFMGSTLLKATPAVLADQGQPE
Cocal_virus       61  HEKFHFSVKLTVQSNRPFRTYDDVVAAVSNWDHMYIGMAGKRPFYKVLAFLASTHLRATPAVLADQGHPE
Alagoas_virus     61  YEKFHFSIKLTVQSNRPFRSCEDAMIAVSNWDHMYIGLAGKRPFYKVLALLGSTLLKATPAVLADQGHPE
Carajas_virus     61  YEKFKFSVKITLRTNRPLRTYDDVCQILSKWDAMYVGMMGKRPFYKVLVLIGSSHLQATPAILSDRGQPE
VSV_New_Jersey    61  YEKFRFMLKMTVRSNKPFRSYDDVTAAVSQWDNSYIGMVGKRPFYKIIAVIGSSHLQATPAVLADLNQPE
Chandipura_virus  64  TLKLQYKCVVNINAINPFRDFREAISAMQFWEADYSGYIGKKPFYRAIILHTARQLKTSNPGILDRGVVE
Piry_virus        64  TLKLQYKCVLQVRSESPFTSYLDAVDNVANWEKQYNGFSGKKPFYRAVMVRAVQAMKANPMSLQDGRSPE
Isfahan_virus     61  SLKIQYKCSLQVRAETPFTSFNDAARCISLWETDYRGYAGKKPFYRLLMLIATKKLRAAPMSLMDGNRPE
consensus>50          .eKf.%..kmtvrsn.Pfr.y.#...avs.W#..YiGm.GKrPFYkil....s..$.atpavl.D.g.pE

M2_protein

VSV_Indiana      131  YHTHCEGRAYLPHRMGKTPPMLNVPEHFRRPFNIGLYKGTIELTMTIYDDESLEAAPMIWDHFNSSKFSD
Morreton_virus   131  YHAHCEGRAYLPHRLGPTPPMLNVPEHFRRPFNIGLFRGTIDLTMTLHDDESLEAAPMIWDHFNASRVTD
Maraba_virus     131  YHAHCEGRAYLPHRLGPTPPMLNVPEHFRRPFNIGLFRGTIDITLVLFDDESVDSAPVIWDHFNASRLSS
Cocal_virus      131  YHAFCEGRAYLPHRLGATPPMLNVPEHFRRPFHIGLYRGTVDLTVTLFDDESVDSANMIWDHFNSSRSYN
Alagoas_virus    131  YHAFCEGRAYLPHRLGPTPPMLNVPEHFRRPFHIGLYRGTVDLTLTLYDDESTESAPVIWDYINSSRVKN
Carajas_virus    131  YHMYLEDRGFIAHRLGLTPPMLSGPESFRRPFHVGLYRGTIDITVNLMDDESTESAPQVWDHFNTRYVNH
VSV_New_Jersey   131  YYATLTGRCFLPHRLGLIPPMFNVQETFRKPFNIGLYKGTLDFTFTVSDDESNEKVPHVWDYMNPKYQSQ
Chandipura_virus 134  YHATTQGRALVFHSLGPSPSMMFVPETFTREWNILTNKGTINVKIWLGETDTLSELEPILNPVNFRDDRE
Piry_virus       134  YTSEIEGRCLVFHSLGHIPPMMYMCEQFTRDWSGRRNQGIVNVKIWVGVTDTLDNLDQIFDPKKHFSEEE
Isfahan_virus    131  YSSMIQGQSIVHHSLGVIPPMMHVPETFTREWNLLTNKGMITAQIWLGITDVVDDLNPLINPALFSDEKE
consensus>50          Yh...egr.y..Hr$G..PpMl.vpE.Frrpf.igly.Gtid.t..l.dd#s.e....iw#..n.....e

M2_protein

VSV_Indiana      201  FREKALMFGLIVEKKASGAWVLDSISHFK
Morreton_virus   201  FPEKALLFGLIVEKKATGAWILDSISNFK
Maraba_virus     201  FREKALLFGLILEKKATGNWVLDSISHFK
Cocal_virus      201  MKEKALLFGLILERKATGSWVLDSISNFK
Alagoas_virus    201  LRENALMFGLIVERKATGSWVLDSISHFK
Carajas_virus    201  FLEHAKRFGLVLSKKPGGGWILDQ.AVCA
VSV_New_Jersey   201  IQQEGLKFGLILSKKATGTWVLDQLSPFK
Chandipura_virus 204  MIEGAAIMGLEIKKQKDNTWLISK.SH..
Piry_virus       204  MLSAATILGLEVKKSSDNNYIISK.SY..
Isfahan_virus    201  MTLTSQMFGLELKKRNDNTWLISK.SY..
consensus>50          ..e.a..fGLi..kk..g.wvld..s...
```
