# Supplementary material for: Visualizing molecular interactions that determine assembly of a bullet-shaped vesicular stomatitis virus particle
Source: Nat Commun. 2022 Aug 15;13:4802. doi: 10.1038/s41467-022-32223-1 (PMC9378655; doi:10.1038/s41467-022-32223-1)
Supplement: Supplementary file 7 — Reporting Summary [file 41467_2022_32223_MOESM7_ESM.pdf]

nature portfolio

Corresponding author(s): Simon Jenni, Stephen C. Harrison

Last updated by author(s): Jul 11, 2022

Reporting Summary

Nature Portfolio wishes to improve the reproducibility of the work that we publish. This form provides structure for consistency and transparency in reporting. For further information on Nature Portfolio policies, see our Editorial Policies and the Editorial Policy Checklist.

Statistics

For all statistical analyses, confirm that the following items are present in the figure legend, table legend, main text, or Methods section.

n/a	Confirmed	
☐	☒	The exact sample size (*n*) for each experimental group/condition, given as a discrete number and unit of measurement
☐	☒	A statement on whether measurements were taken from distinct samples or whether the same sample was measured repeatedly
☒	☐	The statistical test(s) used AND whether they are one- or two-sided<br>*Only common tests should be described solely by name; describe more complex techniques in the Methods section.*
☒	☐	A description of all covariates tested
☒	☐	A description of any assumptions or corrections, such as tests of normality and adjustment for multiple comparisons
☐	☒	A full description of the statistical parameters including central tendency (e.g. means) or other basic estimates (e.g. regression coefficient) AND variation (e.g. standard deviation) or associated estimates of uncertainty (e.g. confidence intervals)
☒	☐	For null hypothesis testing, the test statistic (e.g. *F*, *t*, *r*) with confidence intervals, effect sizes, degrees of freedom and *P* value noted<br>*Give P values as exact values whenever suitable.*
☒	☐	For Bayesian analysis, information on the choice of priors and Markov chain Monte Carlo settings
☒	☐	For hierarchical and complex designs, identification of the appropriate level for tests and full reporting of outcomes
☒	☐	Estimates of effect sizes (e.g. Cohen's *d*, Pearson's *r*), indicating how they were calculated

Our web collection on statistics for biologists contains articles on many of the points above.

Software and code

Policy information about availability of computer code

Data collection	SerialEM (v3.7.1-64bit): Collection of images and movies from electron microscopes.<br>Reference: Mastronarde, D. N. Automated electron microscope tomography using robust prediction of specimen movements. J. Struct. Biol. 152, 36-51, doi:10.1016/j.jsb.2005.07.007 (2005).
Data analysis	MotionCor2 (v1.2.6): Alignment of movie frames.<br>Reference: Zheng, S. Q. et al. MotionCor2: anisotropic correction of beam-induced motion for improved cryo-electron microscopy. Nat. Methods 14, 331-332, doi:10.1038/nmeth.4193 (2017).<br><br>EMAN2 (v2.22, https://blake.bcm.edu/emanwiki/EMAN2): EMAN2 is a broadly based greyscale scientific image processing suite with a primary focus on processing data from transmission electron microscopes.<br>Reference: Bell, J. M., Chen, M., Baldwin, P. R. & Ludtke, S. J. High resolution single particle refinement in EMAN2.1. Methods 100, 25-34, doi:10.1016/j.ymeth.2016.02.018 (2016).<br><br>mag_distortion_correct (v1.00): Correction of magnification distortion.<br>Reference: Grant, T., and Grigorieff, N. (2015). Automatic estimation and correction of anisotropic magnification distortion in electron microscopes. J. Struct. Biol. 192, 204-208.<br><br>Relion (v3.0.8): Cryo-EM data processing software.<br>Reference: Scheres, S. H. RELION: implementation of a Bayesian approach to cryo-EM structure determination. J. Struct. Biol. 180, 519-530, doi:10.1016/j.jsb.2012.09.006 (2012).<br><br>Gctf (v1.06, https://www.mrc-lmb.cam.ac.uk/kzhang/Gctf/): CTF parameter estimation from electron micrographs.

Reference: Zhang, K. Gctf: Real-time CTF determination and correction. J. Struct. Biol. 193, 1-12, doi:10.1016/j.jsb.2015.11.003 (2016).

Frealign (v9.11): Cryo-EM data processing software.
Reference: Grigorieff, N. (2016). Frealign: An Exploratory Tool for Single-Particle Cryo-EM. Methods Enzymol. 579, 191-226.

cisTEM (refine3d version 1.01, reconstruct3d version 1.02, https://cistem.org): cisTEM is user-friendly software to process cry-EM images of macromolecular complexes and obtain high-resolution 3D reconstructions from them.

SPIDER (v24.01): Cryo-EM data processing software.
Reference: Shaikh, T.R., Gao, H., Baxter, W.T., Asturias, F.J., Boisset, N., Leith, A., and Frank, J. (2008). SPIDER image processing for single-particle reconstruction of biological macromolecules from electron micrographs. Nat. Protoc. 3, 1941-1974.

SciPy (v1.8.0): SciPy provides algorithms for optimization, integration, interpolation, eigenvalue problems, algebraic equations, differential equations, statistics and many other classes of problems.
Reference: Virtanen, P., Gommers, R., Oliphant, T.E., Haberland, M., Reddy, T., Cournapeau, D., Burovski, E., Peterson, P., Weckesser, W., Bright, J., et al. (2020). SciPy 1.0: fundamental algorithms for scientific computing in Python. Nat. Methods 17, 261-272.

BioPython (v1.78, https://biopython.org): Biopython is a set of freely available tools for biological computation written in Python by an international team of developers.
Reference: Cock, P. J. et al. Biopython: freely available Python tools for computational molecular biology and bioinformatics. Bioinformatics 25, 1422-1423, doi:10.1093/bioinformatics/btp163 (2009).

ResMap (v1.1.4): Local resolution estimation of cryo-EM maps.
Reference: Kucukelbir, A., Sigworth, F. J. & Tagare, H. D. Quantifying the local resolution of cryo-EM density maps. Nat. Methods 11, 63-65, doi:10.1038/nmeth.2727 (2014).

LocScale (v0.1): LocScale performs local amplitude scaling based on a atomic reference structure.
Reference: Jakobi, A.J., Wilmanns, M., and Sachse, C. (2017). Model-based local density sharpening of cryo-EM maps. Elife 6.

IMOD (v4.9.10, https://bio3d.colorado.edu/imod/): IMOD is a set of image processing, modeling and display programs used for tomographic reconstruction and for 3D reconstruction of EM serial sections and optical sections.
Reference: Mastronarde, D. N. & Held, S. R. Automated tilt series alignment and tomographic reconstruction in IMOD. J. Struct. Biol. 197, 102-113, doi:10.1016/j.jsb.2016.07.011 (2017).

O (v15.0.0): Molecular graphics program, model building.
Reference: Jones, T. A., Zou, J. Y., Cowan, S. W. & Kjeldgaard, M. Improved methods for building protein models in electron density maps and the location of errors in these models. Acta Crystallogr A 47, 110-119 (1991).

PHENIX (v1.17.1-3660, https://www.phenix-online.org): PHENIX is a software suite for the automated determination of molecular structures using X-ray crystallography and other methods.
References: Afonine, P. V. et al. Real-space refinement in PHENIX for cryo-EM and crystallography. Acta Crystallogr D Struct Biol 74, 531-544, doi:10.1107/S2059798318006551 (2018). Afonine, P. V. phenix.mtriage: a tool for analysis and validation of cryo-EM 3D reconstructions. Computational Crystallography Newsletter 8, 25 (2017).

MolProbity (v4.5, http://molprobity.biochem.duke.edu): Molecular structure validation.
Reference: Chen, V. B. et al. MolProbity: all-atom structure validation for macromolecular crystallography. Acta Crystallogr. D Biol. Crystallogr. 66, 12-21, doi:10.1107/S0907444909042073 (2010).

PyMOL (v2.4, The PyMOL Molecular Graphics System, Version 2.1 Schrödinger, LLC): Molecular graphics visualization.
POV-Ray (v3.8.0-alpha.unofficial, www.povray.org): The Persistence of Vision Raytracer is a high-quality, Free Software tool for creating stunning three-dimensional graphics. The source code is available for those wanting to do their own ports.

matplotlib (v3.2.1, https://matplotlib.org): Matplotlib is a comprehensive library for creating static, animated, and interactive visualizations in Python.
Reference: Hunter, J. D. Matplotlib: A 2D graphics environment. Comput. Sci. Eng. 9, 90-95 (2007).

MAFFT (v7.471, https://mafft.cbrc.jp/alignment/software/): MAFFT is a multiple sequence alignment program for unix-like operating systems.
Reference: Katoh, K., Misawa, K., Kuma, K. & Miyata, T. MAFFT: a novel method for rapid multiple sequence alignment based on fast Fourier transform. Nucleic Acids Res. 30, 3059-3066, doi:10.1093/nar/gkf436 (2002).

ESPript (v3.0, http://espript.ibcp.fr/ESPript/ESPript/): 'Easy Sequencing in PostScript', is a program which renders sequence similarities and secondary structure information from aligned sequences for analysis and publication purpose.
Reference: Gouet, P., Courcelle, E., Stuart, D. I. & Metoz, F. ESPript: analysis of multiple sequence alignments in PostScript. Bioinformatics 15, 305-308, doi:10.1093/bioinformatics/15.4.305 (1999).

DSSP (v2.2.0): Protein secondary structure assignment.
Reference: Kabsch, W., and Sander, C. (1983). Dictionary of protein secondary structure: pattern recognition of hydrogen-bonded and geometrical features. Biopolymers 22, 2577-2637.

PISA (v2.1.2): Protein Interactions, Surfaces and Assemblies (PISA).

Reference: Krissinel, E., and Henrick, K. (2007). Inference of macromolecular assemblies from crystalline state. J Mol Biol 372, 774-797.

ConSurf (v2016, https://consurf.tau.ac.il): Positional conservation scores from the multiple sequence alignments.
Reference: Ashkenazy, H., Abadi, S., Martz, E., Chay, O., Mayrose, I., Pupko, T., and Ben-Tal, N. (2016). ConSurf 2016: an improved methodology to estimate and visualize evolutionary conservation in macromolecules. Nucleic Acids Res 44, W344-350.

CODE AVAILABILITY

The software programs used to generate and analyze the data of this study are publicly available. Custom-written C shell and Python scripts used to run the programs and analyze data are available from the corresponding authors on reasonable request.

For manuscripts utilizing custom algorithms or software that are central to the research but not yet described in published literature, software must be made available to editors and reviewers. We strongly encourage code deposition in a community repository (e.g. GitHub). See the Nature Portfolio guidelines for submitting code & software for further information.

Data

Policy information about availability of data

All manuscripts must include a data availability statement. This statement should provide the following information, where applicable:

- Accession codes, unique identifiers, or web links for publicly available datasets
- A description of any restrictions on data availability
- For clinical datasets or third party data, please ensure that the statement adheres to our policy

DATA AVAILABILITY

The cryo-EM maps generated in this study have been deposited in the Electron Microscopy Data Bank under accession codes EMD-26603 (local reconstruction) [https://www.emdataresource.org/EMD-26603] and EMD-26602 (N = 38.5 helical reconstruction) [https://www.emdataresource.org/EMD-26602]. The refined atomic coordinates generated in this study have been deposited in the Protein Data Bank under accession codes PDB-ID 7UML (local reconstruction) [https://www.rcsb.org/structure/7UML] and PDB-ID 7UMK (N = 38.5 helical reconstruction) [https://www.rcsb.org/structure/7UMK]. The previously published VSV structures used in this study are available in the Protein Data Bank under accession codes PDB-IDs 2GIC [https://www.rcsb.org/structure/2GIC], 5UK4 [https://www.rcsb.org/structure/5UK4], and 1LG7 [https://www.rcsb.org/structure/1LG7]). The VSV protein sequences used in this study to prepare the sequence alignments (shown in Supplementary Data 1–3) are available in the UniProt67 database under the following accession codes. The accession codes for the N protein sequences are: VSV_Indiana (P03523) [https://www.uniprot.org/uniprotkb/P03523/entry], vesicular stomatitis virus (Indiana strain); Morreton_virus (A0A0D3R1D1) https://www.uniprot.org/uniprotkb/A0A0D3R1D1/entry], Morreton vesiculovirus; Maraba_virus (F8SPF5) [https://www.uniprot.org/uniprotkb/F8SPF5/entry], Maraba virus; Cocal_virus (B3FRL0) [https://www.uniprot.org/uniprotkb/B3FRL0/entry], Cocal virus; Alagoas_virus (B3FRL5) [https://www.uniprot.org/uniprotkb/B3FRL5/entry], vesicular stomatitis Alagoas virus; Carajas_virus (A0A0D3R1M8) [https://www.uniprot.org/uniprotkb/A0A0D3R1M8/entry], Carajas virus; VSV_New_Jersey (P16379) [https://www.uniprot.org/uniprotkb/P16379/entry], vesicular stomatitis New Jersey virus; Chandipura_virus (P11211) [https://www.uniprot.org/uniprotkb/P11211/entry], Chandipura virus; Piry_virus (A0A1I9L1X2) [https://www.uniprot.org/uniprotkb/A0A1I9L1X2/entry], Piry virus; Isfahan_virus (P16379) [https://www.uniprot.org/uniprotkb/P16379/entry], Isfahan virus; Rabies_virus (P06025) [https://www.uniprot.org/uniprotkb/P06025/entry], Rabies virus; Mokola_Virus (P0C570) [https://www.uniprot.org/uniprotkb/P0C570/entry], Mokola virus. The accession codes for the M protein sequences are: VSV_Indiana (P03523) [https://www.uniprot.org/uniprotkb/P03523/entry], vesicular stomatitis virus (Indiana strain); Morreton_virus (A0A0D3R1D1) [https://www.uniprot.org/uniprotkb/A0A0D3R1D1/entry], Morreton vesiculovirus; Maraba_virus (F8SPF5) [https://www.uniprot.org/uniprotkb/F8SPF5/entry], Maraba virus; Cocal_virus (B3FRL0) [https://www.uniprot.org/uniprotkb/B3FRL0/entry], Cocal virus; Alagoas_virus (B3FRL5) [https://www.uniprot.org/uniprotkb/B3FRL5/entry], vesicular stomatitis Alagoas virus; Carajas_virus (A0A0D3R1M8) [https://www.uniprot.org/uniprotkb/A0A0D3R1M8/entry], Carajas virus; VSV_New_Jersey (P16379) [https://www.uniprot.org/uniprotkb/P16379/entry], vesicular stomatitis New Jersey virus; Chandipura_virus (P11211) [https://www.uniprot.org/uniprotkb/P11211/entry], Chandipura virus; Piry_virus (A0A1I9L1X2) [https://www.uniprot.org/uniprotkb/A0A1I9L1X2/entry], Piry virus; Isfahan_virus (P16379) [https://www.uniprot.org/uniprotkb/P16379/entry], Isfahan virus. All data are available from the corresponding authors upon reasonable request.

Field-specific reporting

Please select the one below that is the best fit for your research. If you are not sure, read the appropriate sections before making your selection.

☒ Life sciences ☐ Behavioural & social sciences ☐ Ecological, evolutionary & environmental sciences

For a reference copy of the document with all sections, see nature.com/documents/nr-reporting-summary-flat.pdf

Life sciences study design

All studies must disclose on these points even when the disclosure is negative.

Sample size	No explicit sample size calculations were performed to design the cryo-EM studies. The main dataset of the N = 38.5 helical reconstruction in this study had a sample size of 21530 segment images obtained from 18353 micrographs (movies with 50 frames each). This number corresponds to a dataset size generally used in the field cryo-EM reconstructions. The local reconstruction is bases on 2985175 images. The number of particles used for each final map is sufficient to obtain reliable classification and reconstruction results by cryo-EM (Scheres, S. H. RELION: implementation of a Bayesian approach to cryo-EM structure determination. J. Struct. Biol. 180, 519-530, doi:10.1016/j.jsb.2012.09.006 (2012)).
Data exclusions	Bad particle images were manually excluded after 2D classification. This is the generally adopted practice in the cryo-EM field (Scheres, S. H.

	RELION: implementation of a Bayesian approach to cryo-EM structure determination. J. Struct. Biol. 180, 519-530, doi:10.1016/j.jsb.2012.09.006 (2012). Grant, T., Rohou, A. & Grigorieff, N. cisTEM, user-friendly software for single-particle image processing. Elife 7, doi:10.7554, eLife.35383 (2018)). The exclusion criteria were not pre-established, but self-evident from the appearance of the 2D class averages.
Replication	Three cryo-EM datasets were collected from three independently prepared samples and on two different microscopes, and evaluated independently, yielding consistent results. The final calculations presented in this paper are from a single cryo-EM dataset.
Randomization	For statistical validation, cryo-EM datasets were randomly split into two half for calculation of cross correlation coefficients (FSC curves) between two the half sets (see Methods section for details). 2D classification in was started from a randomized class distribution.
Blinding	The analysis was performed on single cryo-EM datasets. Blinding of the cryo-EM analysis work-flow was not feasible. The investigators were not blinded to group allocation during data collection. There was no blinding in structural data analysis, because we are studying a specific virus assembly.

Reporting for specific materials, systems and methods

We require information from authors about some types of materials, experimental systems and methods used in many studies. Here, indicate whether each material, system or method listed is relevant to your study. If you are not sure if a list item applies to your research, read the appropriate section before selecting a response.

Materials & experimental systems

n/a	Involved in the study	
☒	☐	Antibodies
☐	☒	Eukaryotic cell lines
☒	☐	Palaeontology and archaeology
☒	☐	Animals and other organisms
☒	☐	Human research participants
☒	☐	Clinical data
☒	☐	Dual use research of concern

Methods

n/a	Involved in the study	
☒	☐	ChIP-seq
☒	☐	Flow cytometry
☒	☐	MRI-based neuroimaging

Eukaryotic cell lines

Policy information about cell lines

Cell line source(s)	BSR-T7 cells were provided by K. Conzelmann, the last author on the cited J. Virol. article here:<br><br>BSR-T7 cells (PubMed=9847328; DOI=10.1128/JVI.73.1.251-259.1999, Buchholz U.J., Finke S., Conzelmann K.-K. Generation of bovine respiratory syncytial virus (BRSV) from cDNA: BRSV NS2 is not essential for virus replication in tissue culture, and the human RSV leader region acts as a functional BRSV genome promoter. J. Virol. 73:251-259(1999)).
Authentication	The cell line was not authenticated.
Mycoplasma contamination	The cell line was not tested for Mycoplasma contamination.
Commonly misidentified lines (See ICLAC register)	BSR-T7 cells are not listed in the ICLAC register of commonly misidentified cell lines.
